# Disinfecting handheld electronic devices with UV-C in a healthcare setting

**DOI:** 10.1016/j.infpip.2021.100133

**Published:** 2021-02-27

**Authors:** Suzan Cremers-Pijpers, Carsten van Rossum, Mirjam Dautzenberg, Heiman Wertheim, Alma Tostmann, Joost Hopman

**Affiliations:** a Hygiene and Infection Control, Department of Medical Microbiology, Radboudumc Center for Infectious Diseases, Radboud University Medical Centre, Nijmegen, the Netherlands; b Department of Medical Microbiology, Radboudumc, the Netherlands

**Keywords:** UV-C disinfection, Handheld electronic devices, Bacterial contamination

## Abstract

Handheld Electronic Devices (HEDs) play a central role in the hospital environment. However, they can be a vehicle for transmitting (pathogenic) microorganisms. We studied whether disinfection with UV-C light is successful in disinfecting three different HEDs in a clinical setting. Disinfection with UV-C light was performed with the UV-Smart® D25. We took a total of 800 samples on two departments and counted colony forming units. More than half of the baseline measurements were moderately (>10CFU) or highly (>50 CFU) contaminated. Post-disinfection the CFU was 0 in 87% of measurements. We conclude that the UV-Smart® D25 can be used to disinfect non-critical HEDs in clinical healthcare.

## Introduction

Handheld Electronic Devices (HEDs) play a central role in the current hospital environment, providing an accessible and portable method for delivering personalized healthcare. [1] Proper cleaning and disinfection of non-critical HEDs in healthcare is essential for safe patient care. Disinfection protocols in the hospital in current study recommend regular disinfection of HEDs with disinfection wipes based on alcohol (Bacillol® 30), which effectively reduce the load of most microorganisms. However, use of antiseptic wipes is often suboptimal due to insufficient knowledge or perceived importance. Moreover, not all handheld devices are compatible with these antiseptic wipes and therefore these wipes cannot always be used for all electronic devices. A universal approach that addresses these limitations is therefore preferred.

HEDs have also been shown to be a potential vehicle of transmission of (pathogenic) microorganisms. It has been described that up to 96% of healthcare workers (HCWs) do not regularly clean or disinfect their device and pathogenic microorganisms have been found on 9–27% of these devices. [[2], [3], [4]] If disinfection is not performed on at least a daily basis – and ideally after every use – the bacterial burden does not differ from ‘control’ devices that have not been disinfected. [5] Furthermore, good hand hygiene performance alone is not sufficient to reduce the bacterial load on HEDs. It has been shown that up to 94.5% of HEDs were contaminated with pathogens that have also been identified on the corresponding HCW's hands and the potential for cross-contamination between phones and hands is high. [6,7] Additional cleaning of HEDs is therefore required to minimize the rate of infection. Treatment with UV-C light is an option to reduce the risk of transmission of microorganisms.

UV-C light – which generally has a wavelength ranging between 200 and 280nm [8] – possesses strong disinfecting properties, [9,10] whereas it reduces bacterial load on HEDs to a minor fraction of the original load within one minute. [4] UV-C acts through inflicting damage to a pathogen's RNA and DNA, ultimately resulting in issues regarding cell replication and cell-death. [8] Additionally, UV-C light is harmless to HEDs, whereas it has been shown to only be harmful for devices after many hours of continuous exposure, similar to the effect of direct sunlight. [11] Although the disinfection properties of the UV-Smart® D25 have been shown in experimental settings, the effect in a clinical operational setting needs to be explored. We therefore conducted a study to assess whether the UV-Smart® D25 is able to successfully disinfect three different handheld electronic devices in a clinical operational setting.

## Methods

### Study design

**Figure 1:**
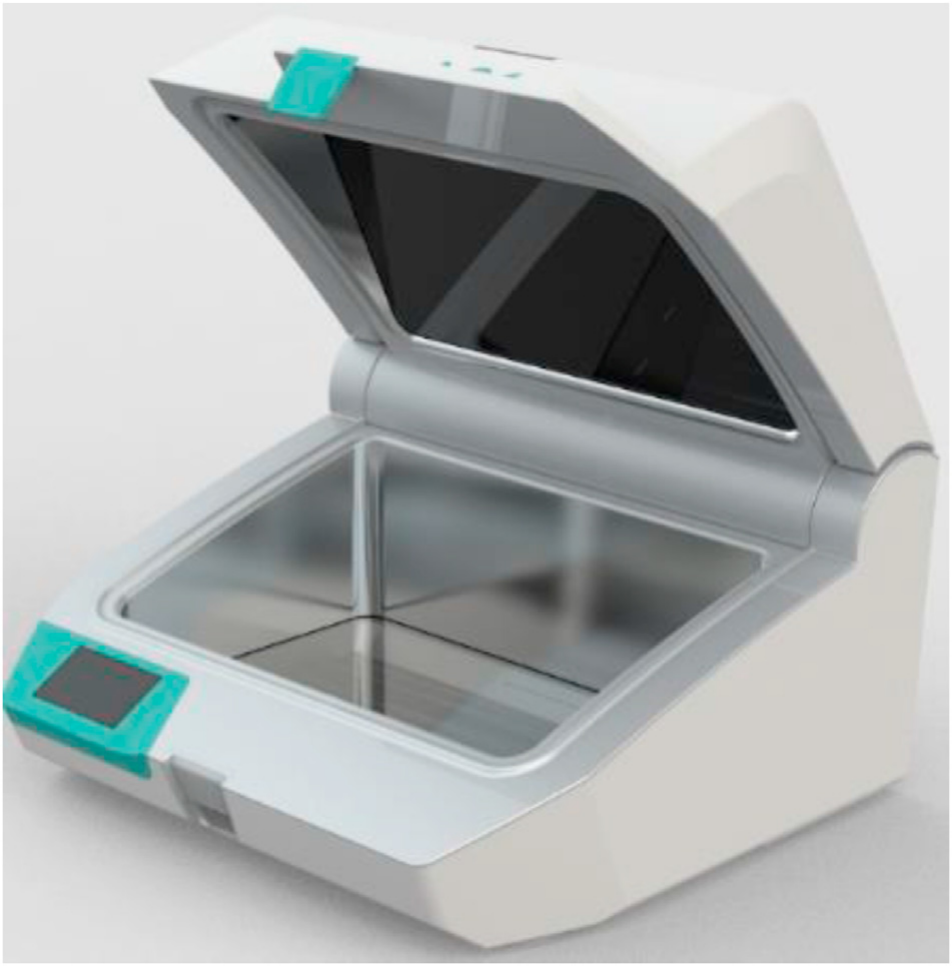
**Photo of the UV-Smart**® **D25**. Outer dimensions of the UV-Smart® are (l x w x h) 470x445x275mm and the chamber dimensions are (l x w x h) 420x265x26mm. The maximum dimension of HEDs that can be disinfected simultaneously are (l x w x h) 380x225x150mm. The UV-Smart® emits UV-C light at a wavelength of 253.7nm.

The UV-Smart® D25 (UV-Smart Company, Delft, The Netherlands) is a mobile UV-C device that can disinfect mobile equipment and non-critical electronic devices in a healthcare setting. [12] Outer dimensions of the UV-Smart® D25 are 470(l)x445(w)x275(h)mm, the chamber dimensions are 420x265x26mm and the maximum dimension of HEDs that can be disinfected simultaneously are 380x225x150mm. The UV-Smart® D25 emits UV-C light at a wavelength of 253.7nm for 25 seconds and ensures 360 degrees exposure of devices placed within using multi-faceted interior reflectors (Figure 1). The UV-Smart® D25 was piloted at the department of Internal Medicine and the Department of Orthopaedics of the Radboud university medical centre in Nijmegen, The Netherlands. These randomly selected adult wards consist of 25 and 20 beds, respectively.

**Figure 2:**
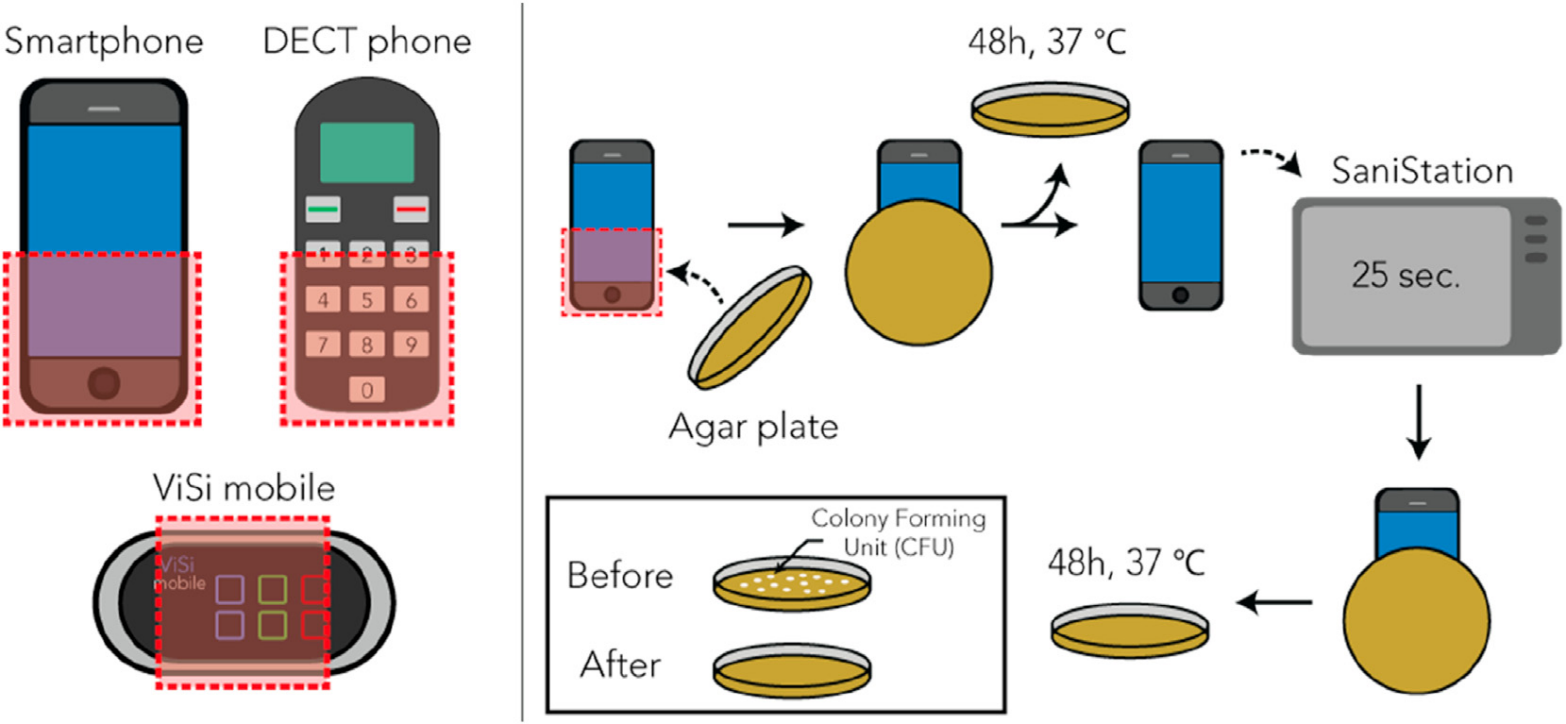
**Schematic representation of the experimental set-up**. Left: The areas of interest are indicated in red for the 3 unique devices: a smartphone (N=100 baseline and post-disinfection), DECT phone (N=200) and ViSi Mobile® (N=100), respectively. Right: The number of colony forming units (CFUs) of a device at baseline and post disinfection using the UV-Smart® D25 were measured using a total count RODAC plates.

The effect of the UV-Smart® D25 was tested on surfaces of three different HEDs that are frequently used in our hospital by doctors, nurses or patients: regular smartphones, DECT (Digital European Cordless Telecommunications) phones (Sonova Nederland B.V., Vianen, the Netherlands), and ViSi Mobiles® (Sotera Wireless, San Diego, USA). All three devices are used for the improvement of patient care. Smartphones (individual use) and the DECT phone (hospital property, used by nurses) enhance communication and administration tasks. The ViSi Mobile® is patient bound and designed for continuous monitoring of vital functions. The effect on bacterial contamination of these devices was assessed by counting the colony forming units (CFUs) on RODAC (Replicate Organism Detection And Counting) plates (Balis laboratiorium, Boven-Leeuwen, the Netherlands). (Figure 2).

The UV-Smart® D25 was positioned at one nursing station and one nurse meeting room. A team of dedicated HCWs on the departments and an Infection Prevention and Control (IPC) trainee were instructed by an IPC professional how to use the UV-Smart® D25, using a user's manual (Appendix A) and a short video created by the department of infection prevention and the manufacturer.

### Data collection

In December 2018 and January 2019, we took 100 samples from a ViSi Mobile® touchscreen and 100 samples from a DECT Phone at the Department of Internal Medicine. In the same time period, we took 100 samples from smartphones of both nurses and doctors and 100 samples from DECT Phones at the Department of Orthopaedics (Table I). From each device, both a baseline and a post-disinfection sample was taken, resulting in a total of 800 measurements (400 baseline and 400 post-disinfection). The trained personnel ensured HEDS were sampled at the most frequently touched areas (Figure 2). Sampling was performed three days per week for four weeks. ViSi Mobiles® and DECT phones were sampled once daily before placement in their designated chargers. Smartphones were sampled throughout the entire day.

**Table I:** Devices sampled and their respective user group for the two departments included in current study

Department	Devices	Amount	User group
Orthopaedics	DECT phones	100	Nurses
	Smartphones	100	Nurses and Doctors
Internal medicine	DECT phones	100	Nurses
	ViSi Mobile®	100	Used for continuous monitoring. Attached to patients, handled by nurses

### Data analysis

The CFU counts per RODAC plate were described as a median (interquartile range, IQR) due to the expected skewed distribution of these values. Median baseline CFU counts were compared between the different devices using a Mann-Whitney U test. Median baseline and post-disinfection CFU counts were compared using a Wilcoxon signed rank test for each of the three devices separately. We chose to display the absolute reduction in CFU since the number of CFU in the baseline measurements was too low to calculate reliable Log-reductions. We discarded RODAC/CFU counts for both the baseline and post-disinfection test if either of the test results could not be used due to contamination or other errors. Disinfection of materials should reduce the number of CFU to below the detection level. The following thresholds for contamination (based on hospital standards) were used: low level of contamination (<10 CFU/RODAC), moderate contamination (10–50 CFU/RODAC) and high level of contamination (≥50 CFU/RODAC).

## Results

In total we conducted 800 measurements, however we had to exclude two smartphone measurements, six DECT phone measurements and four ViSi Mobile® measurements, resulting in a total of 98, 194 and 96 valid results for the smartphones, DECT phones and ViSi Mobile® touchscreens, respectively.

**Figure 3:**
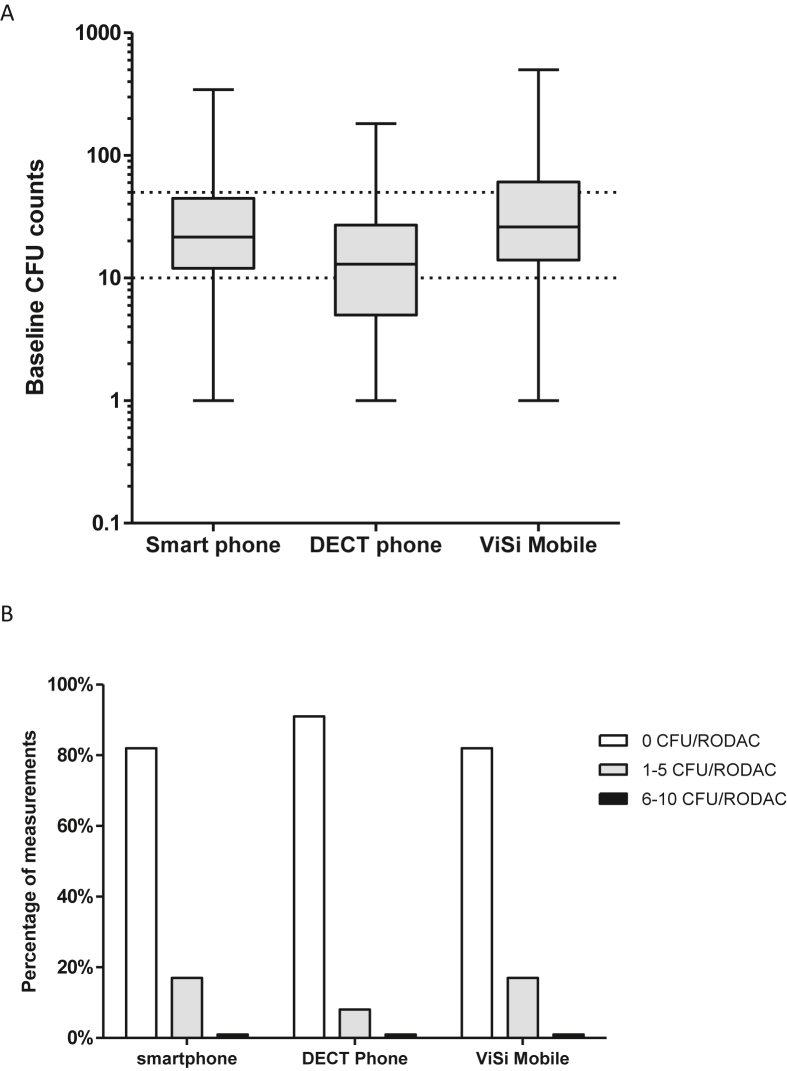
**Pre and post CFU results for all three devices**. **3A.** Baseline CFU results for the smartphones (N=98), the DECT phones (N=194) and the ViSi Mobile® touch screens (N=96). Dotted lines indicate the thresholds for low (<10 CFU) and high (>50 CFU) contamination level. **3B.** Post-disinfection CFU/RODAC counts for the measurements on the smartphones (N=98), DECT phones (N=194) and ViSi Mobile® touchscreens (N=96).

The baseline measurements showed a ‘high level of contamination’ (≥50 CFU) for 19% of the smartphone measurements, 10% of the DECT phone measurements and 31% of the ViSi Mobile® measurements. The median (IQR) baseline CFU counts were 12 CFU/RODAC (12–44) for smartphones, 12 CFU/RODAC (5–27) for the DECT phones and 26 CFU/RODAC (14–60) for the ViSi Mobile® touchscreens (Figure 3). Compared to the DECT phones, the CFU counts were higher on both smartphones (*P*<0.001) and ViSi Mobiles®(*P* <0.001). There was no statistically significant difference in median CFU count between the smartphones and ViSi Mobiles® (*P*=0.20).

### Post-disinfection CFU counts

After UV-C disinfection, overall 87% of measurements had a complete reduction to 0 CFU. For the three device types, 82% of smartphone, 91% of the DECT phone and 82% of the ViSi Mobile® measurements were 0 CFU. This corresponded to a median (IQR) post-disinfection CFU counts of 0 CFU/RODAC (0-0) for all three devices. The CFU counts were only 1–2 CFU/RODAC in 12% of post-disinfection measurements, and the highest post-disinfection CFU count was 7 CFU/RODAC.

The comparison between baseline and post-disinfection CFU counts showed a statistically significant decrease for all three devices (*P*<0.001 for smartphones, DECT phones and ViSi Mobiles®). The overall mean CFU reduction was 97.9%.

## Discussion

This study shows that in a clinical setting, the UV-Smart® D25 reduces the bacterial contamination of smartphones, DECT phones and ViSi Mobiles® to 0 CFU in 87% of measurements. This can be considered a large effect, especially in a context where the average handheld electronic device is contaminated, as was shown by our baseline measurements.

The post UV-C reduction in bacterial load was to be expected, since the effectivity of UV-C light for disinfecting smartphones and tablets has been shown to be more effective than other sanitization methods. [9,13] Others also found (almost) complete reductions in bacterial loads in cycles of 35–60 seconds, whereas Huffman *et al.* stated that no bacterial growth was observed after having inoculated inactive phones and ID-badges for 48 hours with known amounts of bacteria and subsequent disinfection. [4,13].

A bacterial load remained in 13% of devices, however the load was low with mostly 1–2 CFU (12%) and a maximum of 7 CFU. There was no correlation between CFUs after disinfection and the baseline CFU count, which indicates that the performance of the D25 is not dependent on the number of contaminants present pre-disinfection. We hypothesize that the non-uniform distribution of the UV-C light in combination with the time of exposure could explain the partly disinfected surfaces. In addition, previous research showed that the mortality rate of microorganisms using UV-C light is heavily dependent on exposure time. [14] Careful evaluation and optimization of UV-C exposure times should therefore be conducted to elucidate the effect of UV-C exposure time on the rate of disinfection.

The remaining bacterial load can also be caused by reduced susceptibility of certain microorganisms to UV-C light. For example, some – mostly spore-forming – pathogens (such as C. difficile and B. subtilis) appear to be more resilient than others. [15,16] However, UV-C light has not only been shown to be very effective against a wide variety of (susceptible) pathogens (including MRSA, norovirus and VRE) [17,18] but has also been shown to eradicate spore-forming microorganisms such as C. difficile in controlled environments (e.g. the UV-Smart® D25). [12,18,19] Even though these “positive” results appear to contrast with the aforementioned studies, the results of the “negative” studies are mainly based on infection rates after disinfection of entire rooms with so-called “towers” and effectivity of these devices is very susceptible to time, dose, distance and the presence of shadows. [16].

Furthermore, soil may also have affected penetration of UV-C light, exposing bacteria to a lower UV-C dose. In ideal circumstances, HCWs should rid their device of contaminants prior to UV-C disinfection. This however, is often not the case in clinical practice due to a lack of time, knowledge or perceived importance. To mimic real-world scenarios, we did not clean HEDs prior to UV-C disinfection and still observed a reduction to 0 CFU in most measurements. Similar to our results, others have also shown that bacterial load reduction is only slightly reduced in the presence of soil. [18,19] Moreover, we regard disinfection with UV-C light as a complementary procedure to reduce bacterial load (and possibly spread of bacteria) on HEDs. The standard moments at which HEDs should be disinfected are not replaced by UV-C disinfection and therefore we do not believe that limited cleaning prior to UV-C disinfection is a threat to hospital safety.

Cleaning and disinfection of rooms could have affected our results, because clean surfaces reduce contamination of HEDs. To limit the spread of microorganisms, rooms are cleaned and disinfected on a daily basis and healthcare workers should abide by the WHO moments of hand hygiene. HEDs should ideally be disinfected after use in a patient room, but at least on a daily basis. However, although disinfection should leave little to no microorganisms on a surface, spread of microorganisms is inevitable and continuous exchange between microorganisms of a patient, its environment and the hands of HCWs is imminent. [2,3,6,7]. To account for varying contamination of HEDs, we took pre-disinfection samples. Furthermore – as mentioned earlier – we did not observe a correlation between pre- and post-disinfection.

In current study, RODAC plates were used instead of the cotton swabs. RODAC plates were selected based on their rich growth-medium suitable to judge the level of surface-contamination. We aimed to assess the amount of growth rather than determination of species, whereas we were primarily interested in microorganisms often identified on surfaces of healthcare settings (e.g., skin flora) that easily grow on these media and less in very specific organisms that are rarely found on such surfaces (e.g., S. pneumoniae and Haemophilus spp). Furthermore, RODAC plates also reduced variation between researchers by allowing the sampled surface to be identical (pressure and location) for every HED tested, whereas swabs can result in heterogeneous sampling. Lastly, we did not use any neutralizing agents on our RODAC plates to inactivate disinfecting residues for two reasons: 1) UV-C light does not leave residues that must subsequently be neutralized and 2) we believe that residues left from previous disinfection cycles (e.g., with wipes) have been inactivated and did not affect growth since the baseline measurements showed extensive growth for all devices.

A limitation of this study was that we only included two departments. It would be interesting to repeat this study in different departments in a multi-centre study. We have only tested three specific handheld electronic device types. Tablets have not been assessed, however, they are becoming more apparent in patient rooms as patient-bound devices. Other noncritical devices to be considered include stethoscopes, blood pressure machines, glucose machines or scissors. Moreover, we have only sampled over a period of twelve non-consecutive days, during which ViSi Mobiles® and DECT phones were sampled after use and smartphones were sampled during the entire day. This structured sampling over a period of only four weeks, may have affected the number and type of bacteria present on HEDs. However, since this study primarily focusses on bacteria, we do not believe that seasonality or time-of-day significantly affects the type and amount of bacteria. Furthermore, the methods used in this study were not-suited for detection of viable but non culturable (VBNC) bacteria and therefore we did not take into account these strains. As a result, optimized screening methods [20] that allow detection of VBNC's should be used to address this type of contamination in more depth. Lastly, we did not perform further determination of bacterial species. However, given the pragmatic approach of current study in combination with the convincing results, we believe that our outcome measure sufficiently answers our research question and therefore justifies not performing further determination.

A challenge for implementation of a UV-Smart® D25 would be to decide on a suitable and feasible frequency of use. Even though bacterial loads get below the detection limit directly after disinfection by UV-C, recontamination via hands and other -often contaminated-hospital environments occurs relatively quickly, returning to pre-disinfection levels within 48 hours. [13] This study suggest that a daily or twice daily disinfecting round with UV-C should result in a consistently low bacterial load. The UV-Smart® D25 can be a valuable addition to the disinfection options in a healthcare setting, provided proper instructions and clear protocols are developed. The 25-second ‘waiting time’ provides a good opportunity for hand disinfection, as was instructed during this pilot.

In conclusion, this study showed that the UV-Smart® D25 can successfully be used to disinfect non-critical handheld electronic devices which are used in the clinical healthcare. However, further applied research is required on the best method to implement the machine in clinical practice to ensure ease of use, high compliance and effective disinfection cycles.

## Ethics approval and consent to participate

Not applicable.

## Availability of data and material

The datasets used and/or analysed during the current study are available from the corresponding author on reasonable request.

## Conflict of interest statement

The authors declare that they have no competing interests.

## Funding

This research did not receive any specific grant from funding agencies in the public, commercial, or not-for-profit sectors. The UV-Smart® D25 machine was kindly provided by the company but no financial support was provided by them, neither did they have a role in the writing, approval and submission of current manuscript.

## Authors contributions

SC: Design, organisation and execution of the project, data collection. CvR: Report writing and literature review. HW: Provided substantial input in the article. AT: Performed data analysis, report writing and finalization. JH: Coordination and supervision of the project, input in article.

## Appendix A Supplementary data

The user’s manual of the UV-Smart® D25 will be supplied as a separate file.
