# Supplementary material for: Disinfecting handheld electronic devices with UV-C in a healthcare setting
Source: Infect Prev Pract. 2021 Feb 27;3(2):100133. doi: 10.1016/j.infpip.2021.100133 (PMC8336058; doi:10.1016/j.infpip.2021.100133)

**UV Smart** D25

**Instructions for use**

**EN**

**English**

# Use of the D25: Quickstart Guide


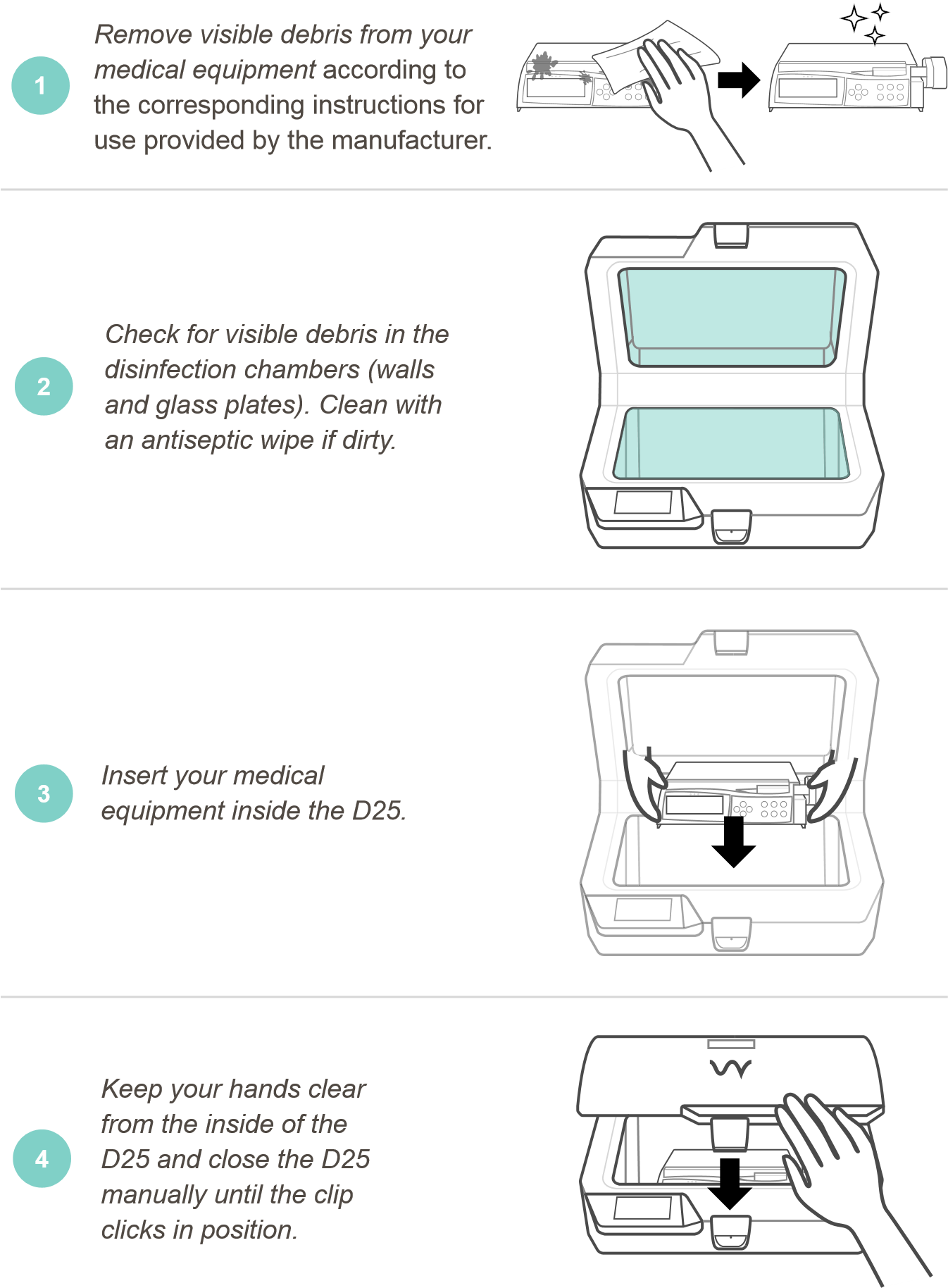


**UV Smart** D25 **|** Instructions for use **|** Revision 8 **EN**

**5**


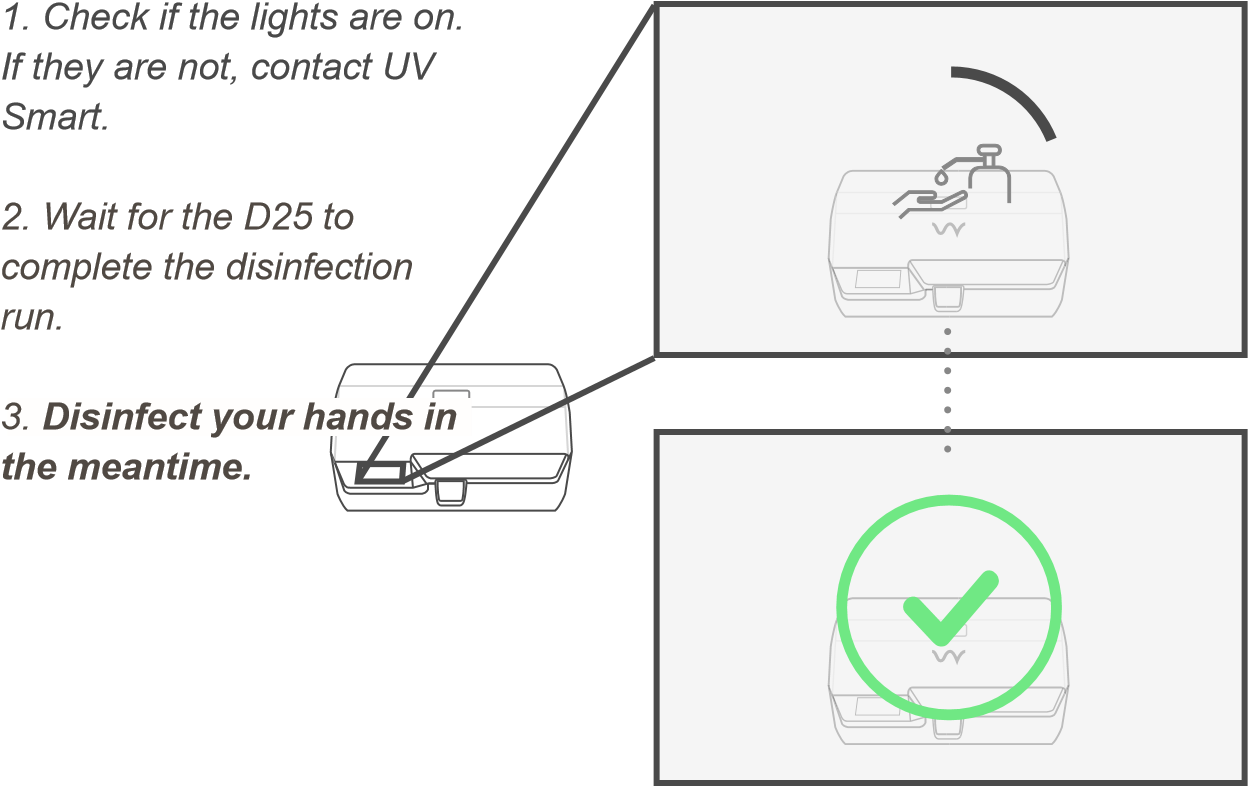


*Your medical equipment has been successfully disinfected and is safe to use. With disinfected hands, take out your medical equipment.*

**6**

**Table of contents**

**Use of the D25: Quickstart Guide**........................................................................*2*

**Introduction** .........................................................................................................*5*

**Intended Use** ........................................................................................................*6*

Intended Use

Intended Medical Indication

Indications for Use

Contra-indications for Use

Intended users

Intended use environment

Claimed performance

**Non-medical D25 Use**..........................................................................................*9*

**Warnings and precautions** .................................................................................*10*

**Product overview** ................................................................................................*14*

Packaging contents

Features and dimensions

**First time use** .......................................................................................................*16*

**Use of the D25** .....................................................................................................*19*

**Interface** ...............................................................................................................*21*

Sounds

Lights

Display

**Maintenance** ........................................................................................................*28*

Cleaning

Transport

Technical safety check

Disposal

**Problem solving** ..................................................................................................*32*

Problem solving

Error codes

Broken glass or lamps

Overheating

**Technical specifications** .....................................................................................*35*

**Symbols** ...............................................................................................................*36*

**Warranty** ...............................................................................................................*37*

**Contact details** ....................................................................................................*37*

Instructions for use

# Introduction

The UV Smart D25 is a software-controlled medical device which provides disinfection of hard, non-porous outer surfaces of non-invasive medical devices using UV-C light.

The UV Smart D25 consists of a sealed disinfection chamber containing ultraviolet germicidal lamps. When in use, the UV-C light that is used for disinfection cannot escape the D25, guaranteeing safe use of the D25.

The germicidal effect of UV-C light will not penetrate dirt, debris and grime and therefore it is required that all medical equipment is cleaned before use according to the corresponding instructions for use provided by the manufacturer to allow the D25 to perform optimally.

The UV Smart D25 should be used by informed personnel. The Instructions for Use should therefore be read prior to operation.

# Intended use

### Intended use

The UV Smart D25 is intended to reduce micro-organism count by at least log-4 on hard, non-porous outer surfaces (glass, plastic, metal) of non-invasive medical devices within 30 seconds by using UV-C light.

The UV Smart D25 is intended for disinfection of the outer surfaces of the following non-invasive medical devices:

▪ Infusion pumps;

▪ Thermometers;

▪ Surveillance monitoring equipment;

▪ Stethoscopes;

▪ Communication systems.

To be disinfected surfaces cannot be shadowed or covered during the disinfection process.

The device does not support or sustain life.

### Intended medical indication

The UV Smart D25 is intended for disinfection of hard, non-porous outer surfaces of non-invasive medical devices and physically able to fit inside the disinfection chamber’s designated volume.

### Indications for Use

The UV Smart D25 can be used in case of the following needs:

▪ Disinfection of common surfaces;

▪ Disinfection within 30 seconds;

▪ Automated disinfection;

▪ Consistent disinfection; ▪ Disinfection on location.

Previously administered, manual disinfection methods can be applied in case of device failure.

**UV Smart** D25 **|** Instructions for use **|** Revision 8 **EN**

### Contra-indications for use

The UV Smart D25 cannot be used in case:

▪ The Indications for Use are not met;

▪ The D25 is damaged;

▪ There is visible debris on medical equipment;

▪ There is visible debris inside the disinfection chamber;

▪ The medical equipment does not consist of hard, non-porous outer surfaces;

▪ The surface is organic, wood or textile;

▪ There is any doubt about how to use the D25.

### Intended users

The UV Smart D25 is suitable for use by any medical professional or laymen working under the supervision of a medical professional. A medical professional is an individual qualified by education, training, licensure or facility privileging who performs a professional service within his/her scope of practice. Medical professionals include doctors, nurses, hospice workers, (emergency) medical technicians and other trained caregivers.

### Intended use environment

The intended use environment of the UV Smart D25 is medical related, meaning; hospitals, medical clinics, nursing home, doctor’s office, etc.

The UV Smart D25 shall be used in the following environmental conditions:

Temperature: -20^o^C – + 50^o^C

Relative Humidity: 20% – 90% (without condensation)

Atmospheric pressure: 700hPa – 1060hPa

**UV Smart** D25 **|** Instructions for use **|** Revision 8 **EN**

## Claimed performance

The required reduction of micro-organisms is based on the EN 14885:2018 to compare it to the state of the art of chemical disinfectants and antiseptics. The following reductions are determined and claimed:

| **Claim** | **Micro-organism** | **Sort and type** | **Log reduction** |
| --- | --- | --- | --- |
| Bactericidal | Staphylococcus aureus | Gram-positive bacteria, vegetative  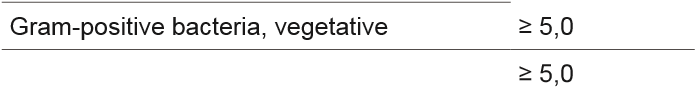Gram-positive bacteria | ≥ 5,0 |
|  | Pseudomonas aeruginosa |  |  |
|  | Enterococcus hirae |  |  |
|  | Escherichia coli | Gram-negative bacteria, Enterobacteriaceae  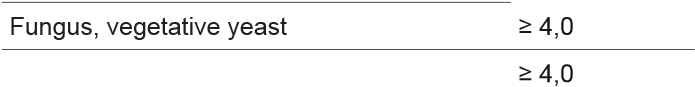Fungus, spore | ≥ 4,0 |
| Fungicidal | Candida albicans |  |  |
|  | Aspergillus brasiliensis |  |  |
| Yeasticidal | Candida albicans | Fungus, vegetative yeast  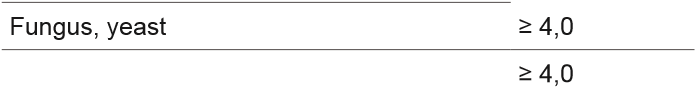Gram-positive bacteria, endospore | ≥ 4,0 |
|  | Candida auris |  |  |
| Sporicidal | Bacillus subtilis |  |  |
| Mycobactericidal | Mycobacterium avium | Gram-positive mycobacteria, vegetative  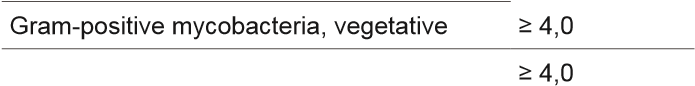Gram-positive mycobacteria, vegetative | ≥ 4,0 |
|  | Mycobacterium terrae |  |  |
| Tuberculocidal | Mycobacterium terrae |  |  |
| Viricidal | Adenovirus type 5, strain Adenoid 75 | Adenoviridae | ≥ 4,0 |

**UV Smart** D25 **|** Instructions for use **|** Revision 8 **EN**

# Non-medical D25 Use

UV-C light can be applied on hard, non-porous surfaces. This also includes surfaces of non-medical devices.

The UV Smart D25 can be used for disinfection of the hard, non-porous outer surfaces (glass, plastics, metal) of the following non-medical devices:

▪ Telephones;

▪ Smartphones;

▪ Tablets;

▪ Badges;

To be disinfected surfaces cannot be shadowed or covered during the disinfection process.

After using the D25 for disinfection of non-medical devices, all visible debris should be removed from the disinfection chambers to make sure disinfection is not impaired. The D25 can be applied for medical use again only if all visible debris is removed from the disinfection chambers.

All information provided on the following pages of this Instruction for Use is also applicable for non-medical use of D25.

# Warnings and precautions

### Warnings

Upon delivery, check that all items are present, without debris and undamaged. Do not use a damaged D25. If you think the D25 is damaged, dirty or items are missing, please contact UV Smart or your local distributor.

Do not place the D25 in a position where a patient or user could come to harm should it fall.

Do not transport the device on a cart if not properly positioned and/or fixed.

Transportation with or affixing to a trolley is at the customer’s own risk

Do not use the USB-port on the back of the device. The port is for UV Smart equipment and maintenance personnel only.

Do not use the D25 in an oxygen enriched environment or in the vicinity of combustible gases (e.g. anesthetics).

The D25 must only be connected to a supply mains with protective earth, to avoid the risk of electric shock.

Visible debris should be removed from the medical equipment surface according to the corresponding instructions for use provided by the manufacturer before placing it inside the D25 for disinfection.

Protect the D25 and the power supply against liquids and moisture.

Follow the instructions as indicated on the device display.

Do not insert medical equipment with working channels.

Do not insert invasive medical equipment.

Do not insert medical equipment made of organic materials.

Only insert medical equipment consisting of hard, non-porous outer surfaces.

**UV Smart** D25 **|** Instructions for use **|** Revision 8 **EN**

Do not insert medical equipment made of elastic materials.

Do not insert medical equipment made of textile materials.

Do not insert sharp objects.

Do not insert humans or animals.

Do not insert flammable substances in the D25 to prevent damage and risk of fire/ explosion.

Do not insert medical equipment larger than the dimensions defined on page 15. This could damage the medical equipment/D25 and/or jeopardize the disinfection performance.

When inserting multiple pieces of medical equipment, they should not overlay and/ or cast shadows on each other.

Medical equipment should not touch the side walls of the disinfection chamber.

Do not close the lid with excessive force.

Do not open the D25 during a disinfection run, unless it is instructed by the display or when strictly necessary because of external factors.

### Precautions

With initial use, the user is required to read the Instructions for Use.

Do not carry the D25 when the lid is in an open position.

Make sure the D25 is properly positioned on a steady surface.

The display is not a touch screen. The display is solely meant to visually instruct the user.

Avoid applying external force in the ‘opening’ direction when the lid is in open position.

Gently place equipment inside the disinfection chamber of the D25 to prevent damage to the D25 and medical equipment.

Always verify if the UV-C lamps switch on when a disinfection run is ongoing.

Do not place any medical equipment on top of the D25 when it is in operation. After a disinfection run the lid opens automatically and might cause damage to the medical equipment or to the D25 itself.

Only clean the D25 following the cleaning instructions on page 28.

### Placement of medical equipment

*The medical equipment should not be placed outside of the middle plane.*

*The medical equipment should not touch the side walls.*

*The medical equipment should not be higher than the indicated edge.*

Instructions for use

### Indications and contra-indications for use

**Indications for Use** The UV Smart D25 can be used in case of the following needs:

Disinfection of common surfaces;


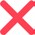

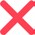

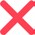

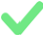

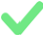

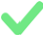

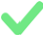

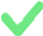


Disinfection within 30 seconds;

Automated disinfection;

Consistent disinfection;

Disinfection on location.
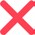


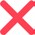


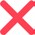

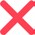


**Contra-indications for use** The UV Smart D25 cannot be used in case:

The Indications for Use are not met;

The D25 is damaged;

There is visible debris on medical equipment;

There is visible debris inside the disinfection chambers; The medical equipment does not consist of hard, non-porous outer surfaces;

The surface is organic, wood or textile; There is any doubt about how to use the D25.

.

# Product overview


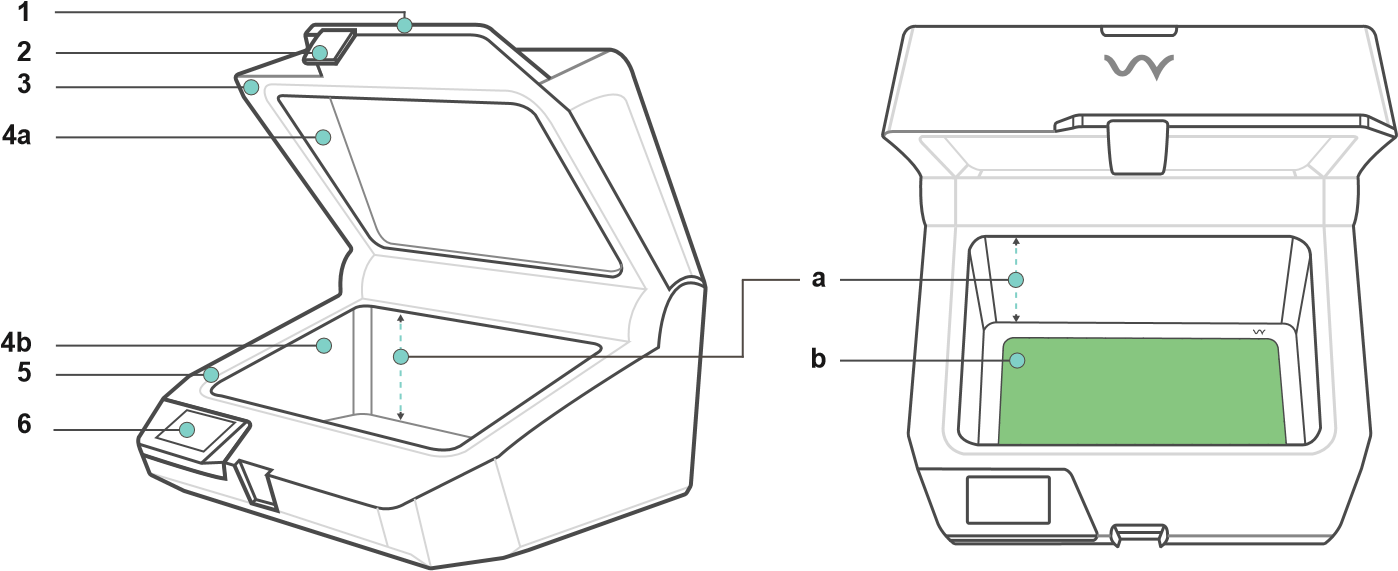
**Packaging content**

**Items**

**1.**

Power cable

**2.**

Instructions for Use

**3.**

D25 Medical Device

**1**

**2**

**3**

## Features and dimensions

Instructions for use

####
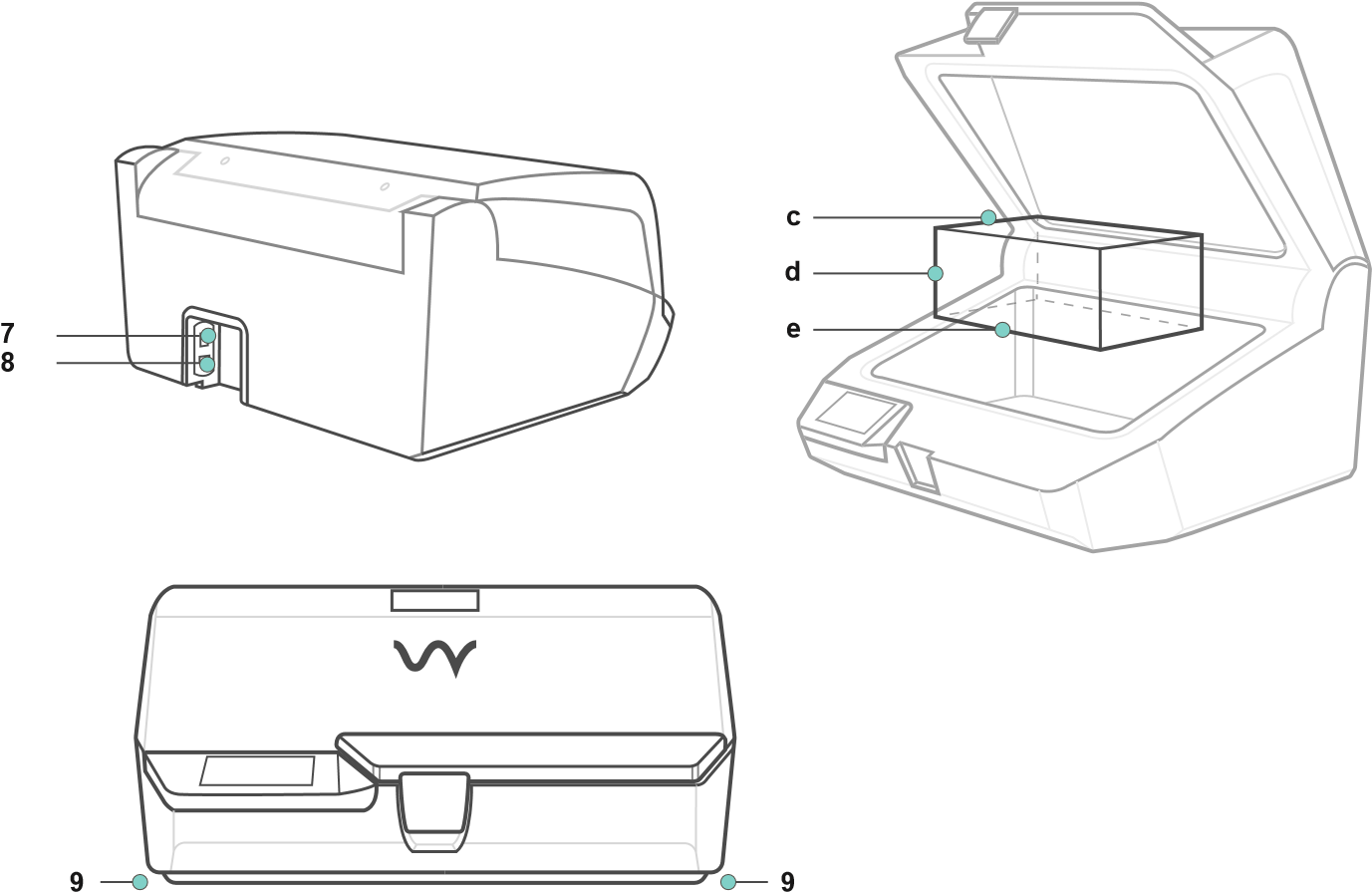
Features

1. Handle
2. Clip
3. Lid

**4a.** Disinfection chamber - top

**4b.** Disinfection chamber - bottom

#### Dimensions

1. Disinfection chamber height

| 1. Maximum medical equipment depth 2. Maximum medical equipment height 3. Maximum medical equipment width | (225mm)  (150mm)  (380mm) |
| --- | --- |
| **UV Smart** D25 **\|** Instructions for use **\|** Revision 8 | **EN** |

1. Disinfection chamber area
2. Top surface
3. Display
4. On/Off-switch
5. Power cable input
6. Carrying features

(150mm)

(225x380mm)

# First time use


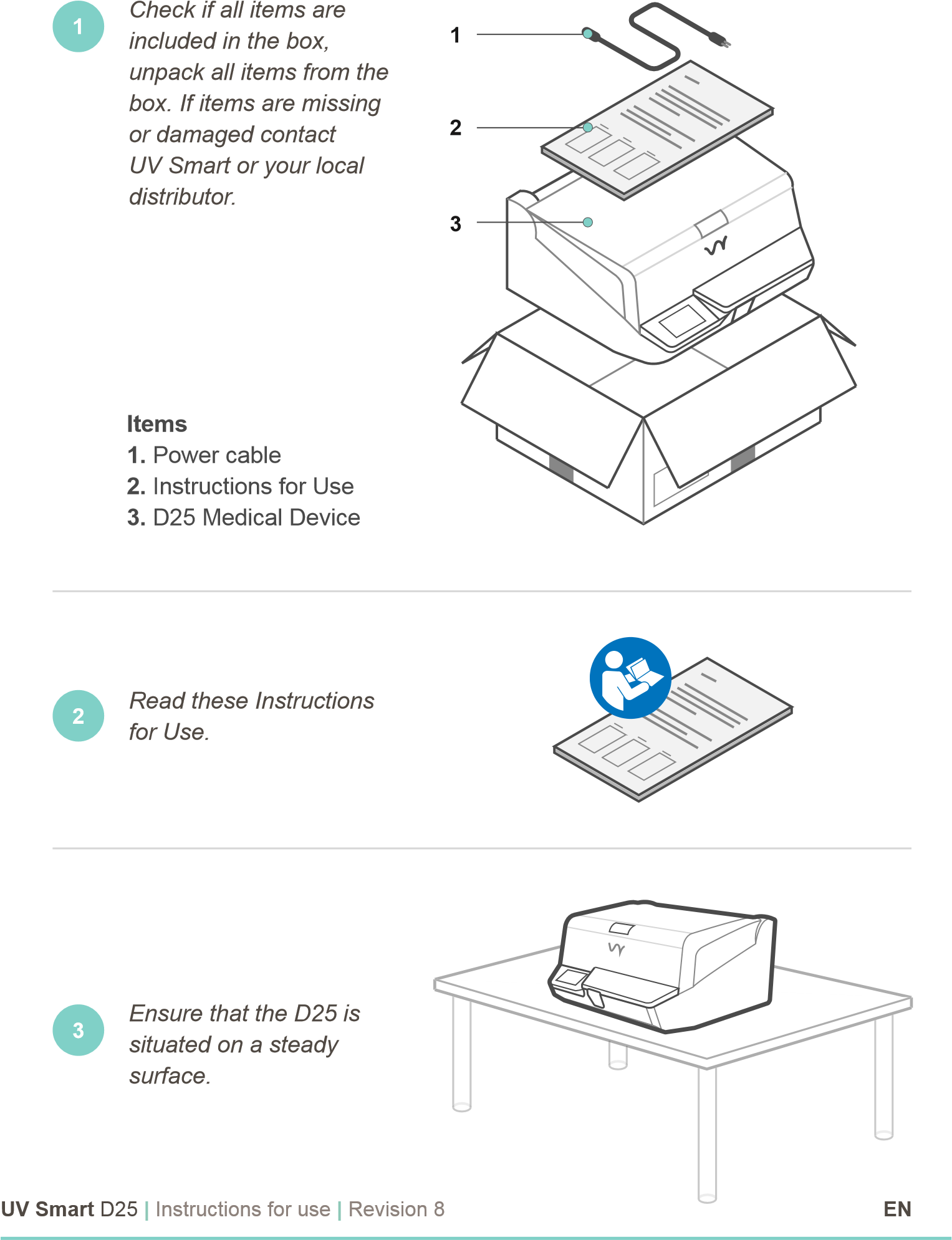


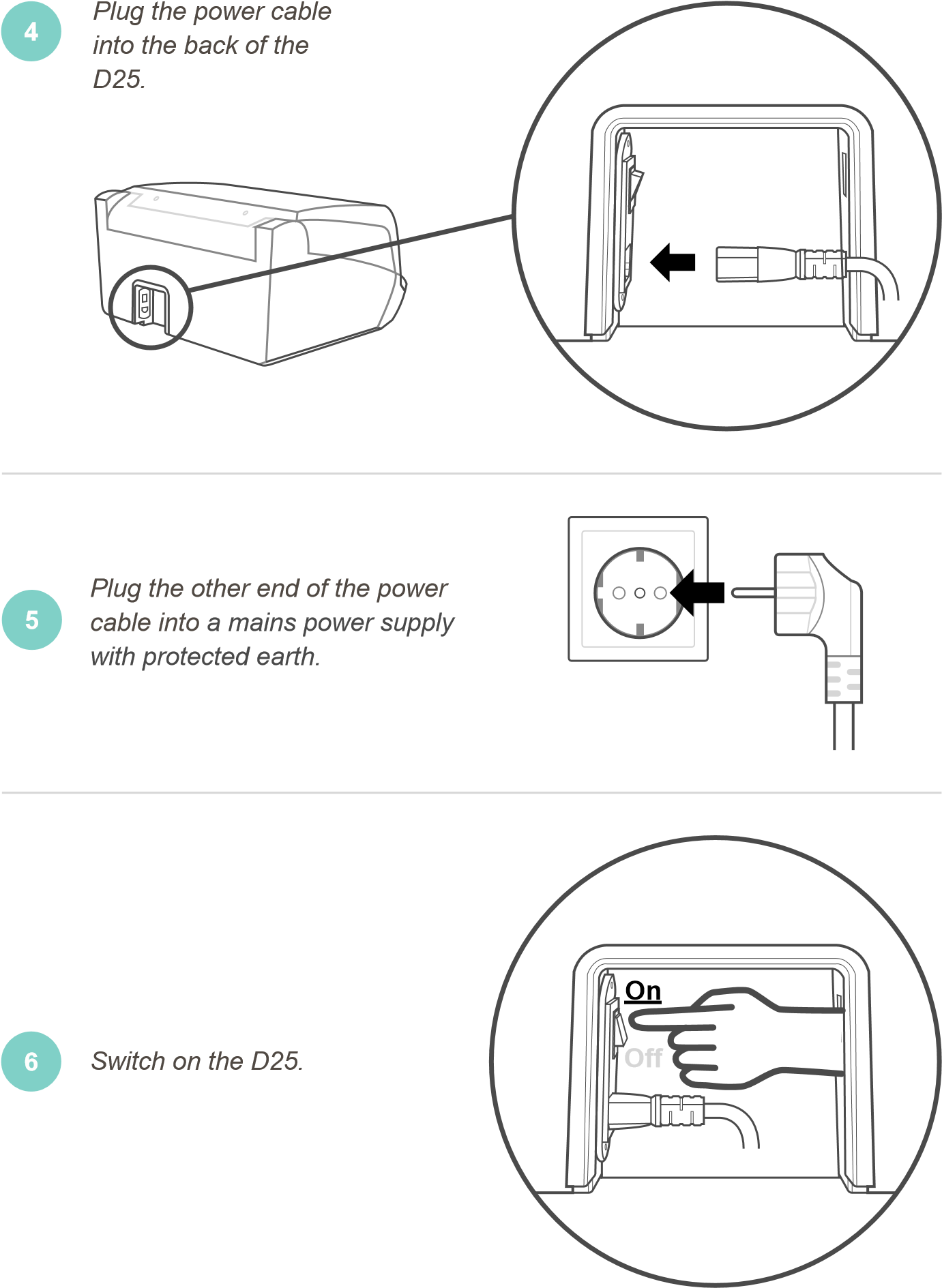


**UV Smart** D25 **|** Instructions for use **|** Revision 8 **EN**

*The D25 is ready for use.*

**7**

# Use of the D25


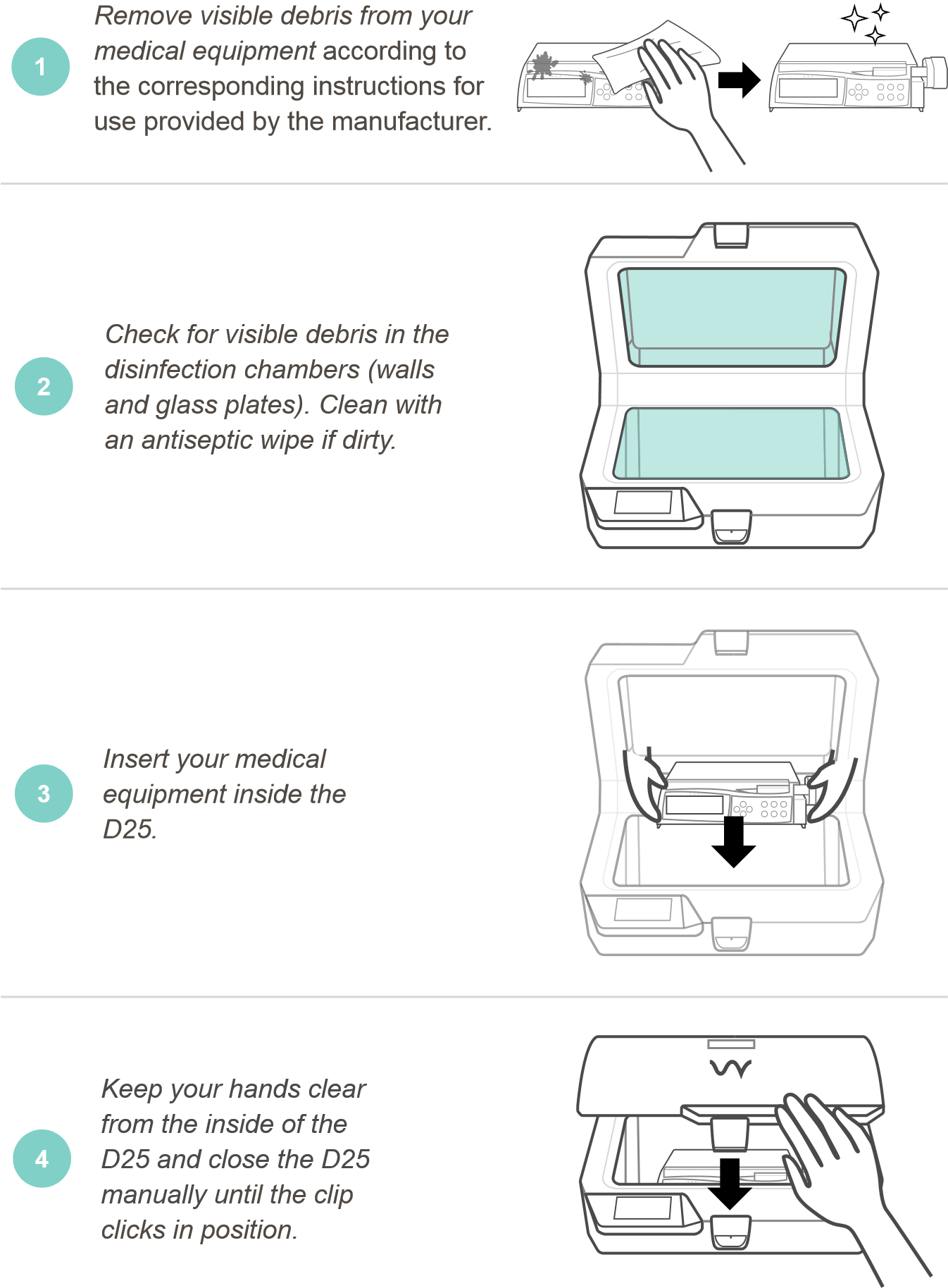


**UV Smart** D25 **|** Instructions for use **|** Revision 8 **EN**

**5**


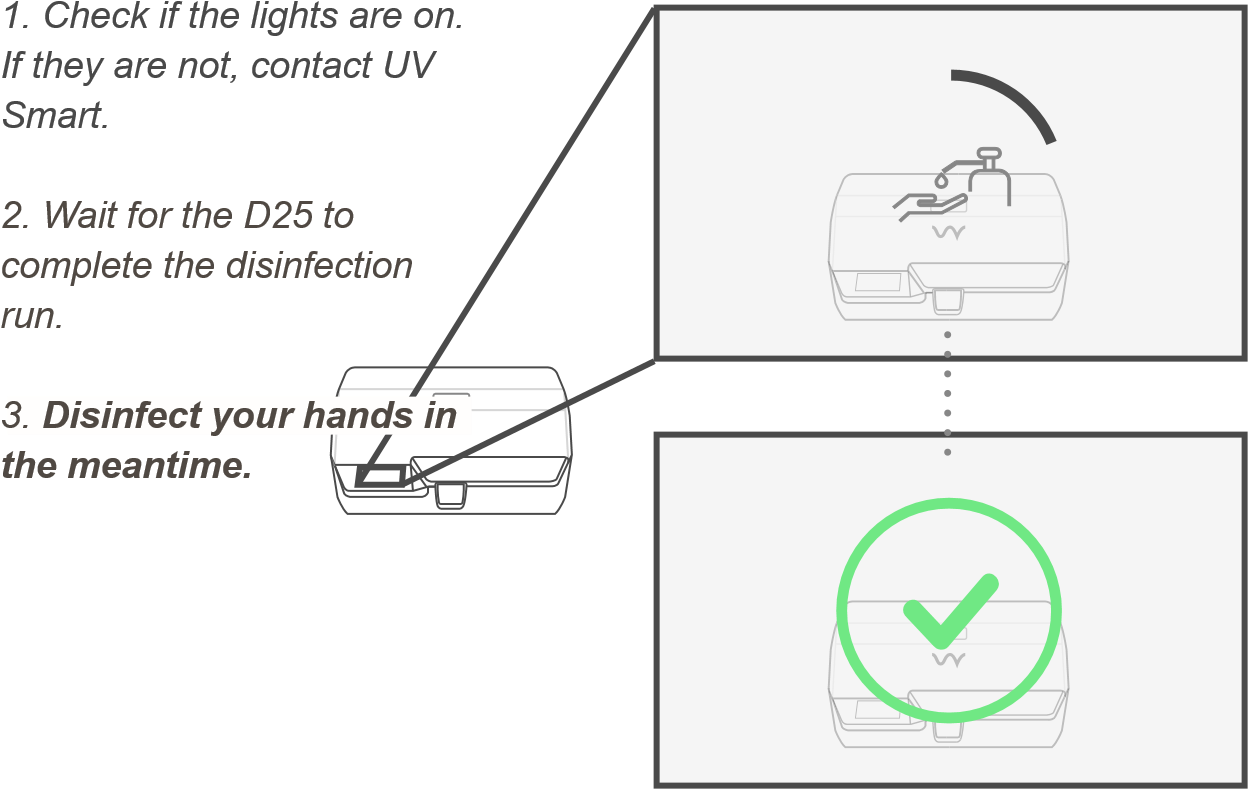


*Your medical equipment has been successfully disinfected and is safe to use. With disinfected hands, take out your medical equipment.*

**6**

# Interface

The D25 interface consists of audio and visual components. Information is communicated to the user through speakers, lights and a display.

**Sound**

**Negative**

**Possible meanings**

The medical equipment has not been disinfected

**Action options**

▪

Check display for instructions


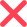


**Sounds**

The lamps are not functioning properly

The opening mechanism is not functioning properly

The medical equipment has been successfully disinfected

**Attention**

The lid is not (properly) closed

**!**

The lid does not open automatically

The D25 needs cleaning

▪ Close the D25 manually ▪ Contact UV Smart

▪ Check display for instructions ▪ Contact UV Smart

▪ Check display for instructions

▪ Contact UV Smart

**Positive**

The D25 is starting up

▪

Check display for instructions


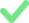


▪ Take out medical equipment

▪ Check display for instructions ▪ Close the lid until the clip clicks in position

▪ Contact UV Smart

▪ Check display for instructions

▪ Open the lid manually ▪ Contact UV Smart

▪ Check display for instructions

▪ Manually clean the D25

| **No Sound**  D25 is ready to be used    ▪  Insert medical equipment  **UV Smart** D25 **\|** Instructions for use **\|** Revision 8 **EN** |
| --- |

▪ Contact UV Smart

## Lights

**Light Possible meanings**

**Red** The medical equipment has not been disinfected

The UV-C lamps are not functioning properly

The opening mechanism is not functioning properly

**White**

D25 is ready to be used

▪

Insert medical equipment

The lid does not open automatically

The D25 needs cleaning

**Action options**

▪ Check display for instructions

▪ Close the D25 manually ▪ Contact UV Smart

▪ Check display for instructions ▪ Contact UV Smart

▪ Check display for instructions ▪ Contact UV Smart

**Green**

**White**

**blinking**

The medical equipment has been successfully

disinfected

The lid is not (properly) closed

▪

Take out medical equipment

▪

Check display for instructions

▪

Close the lid until the clip

clicks in position

▪ Contact UV Smart

▪ Check display for instructions

▪ Open the lid manually ▪ Contact UV Smart

▪ Check display for instructions

▪ Manually clean the D25

▪ Contact UV Smart

### Display

**Startup**

*D25 is starting up, wait until next instruction.*

**A**


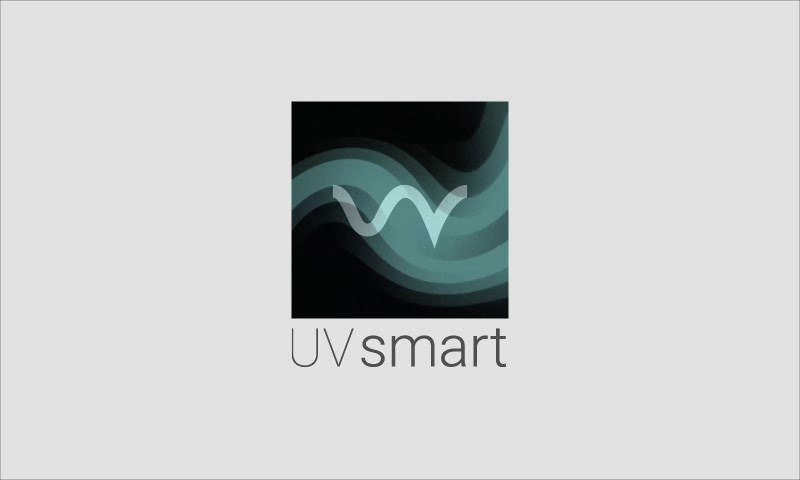


The startup-sound is played.


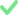


*Close the lid manually.*


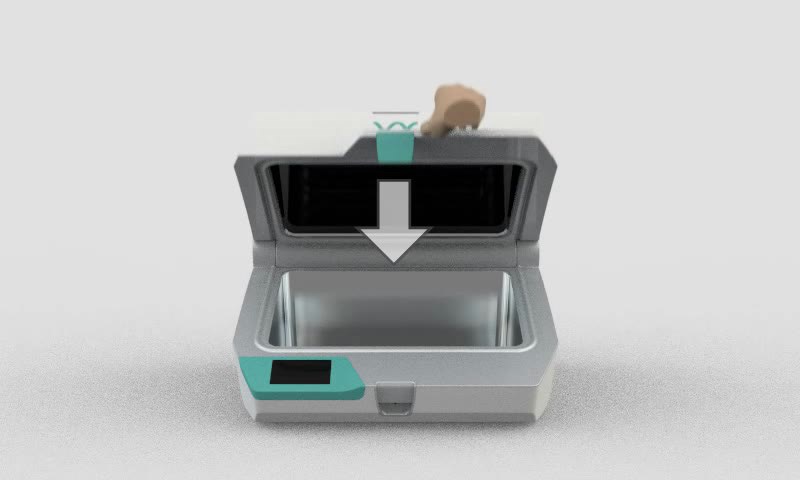


**Close the lid**

**B**

The disinfection chamber lights up white.


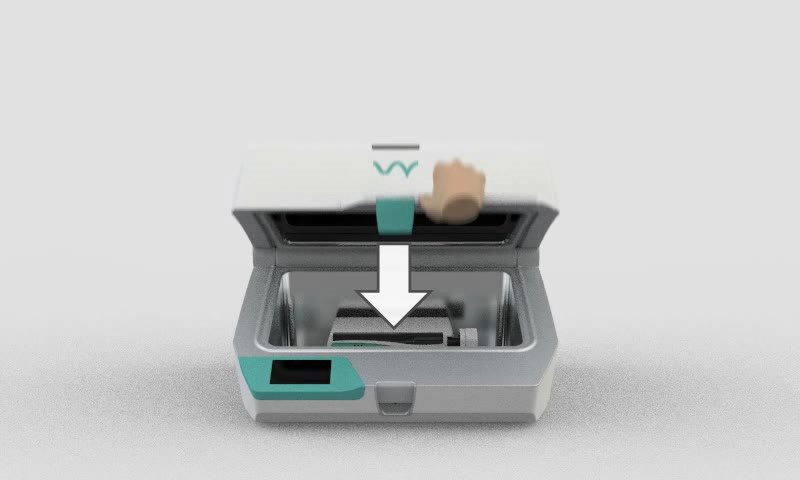

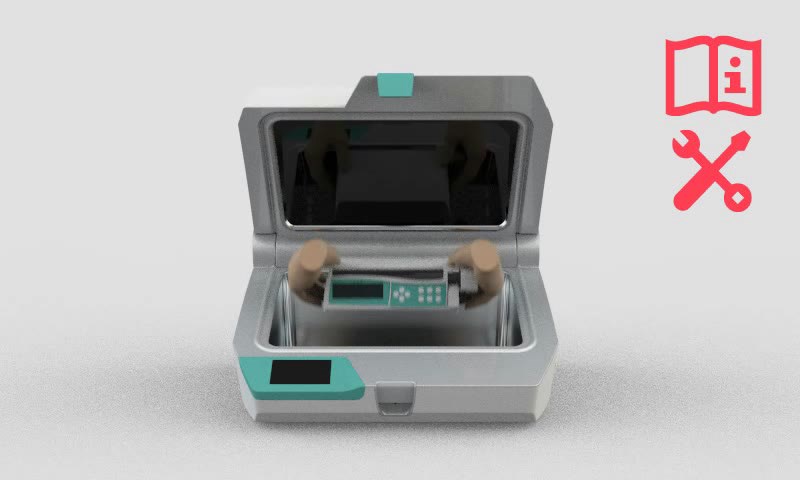

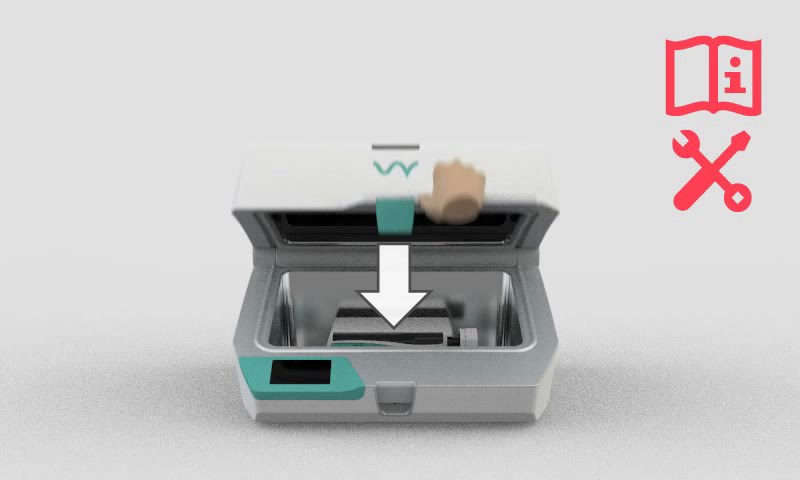

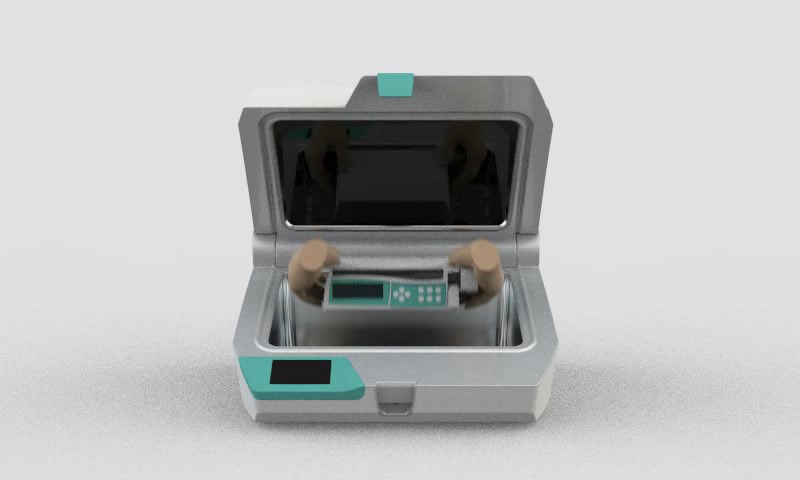


The disinfection

chamber lights up

white.

The disinfection

chamber lights up

white.

*Insert medical equipment*

*and close the lid manually.*

*Insert medical equipment and*

*close the lid manually.*

*D25*

*is still operational but needs*

*maintenance: contact UV Smart*

*or your local distributor (p.37)*

*.*

**Ready for disinfection**

**Ready for disinfection;**

**product needs**

**maintenance**

**C-1**

**C-2**

*The D25 is running*

*the disinfection run.*

*Disinfect your hands in*

*the meantime to avoid*

*recontamination.*

*With disinfected hands,*

*take out the medical*

*equipment.*

**Disinfection ongoing,**

**disinfect hands**

**Disinfection successful**

**D**

**E**


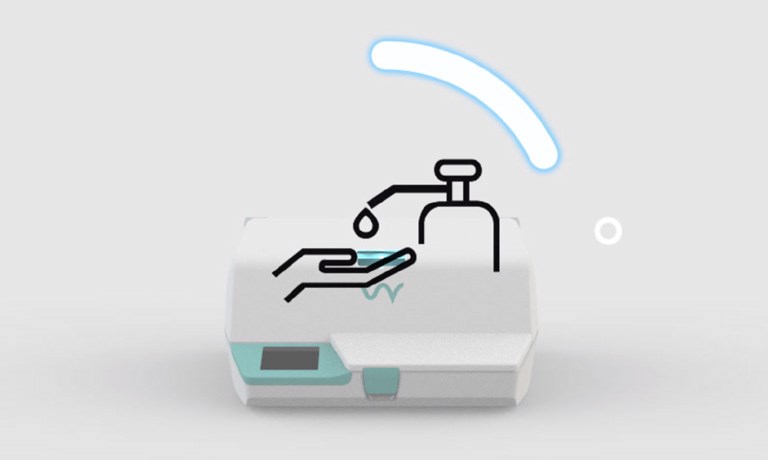


A positive sound is

played.

The disinfection

chamber lights up

green.


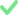

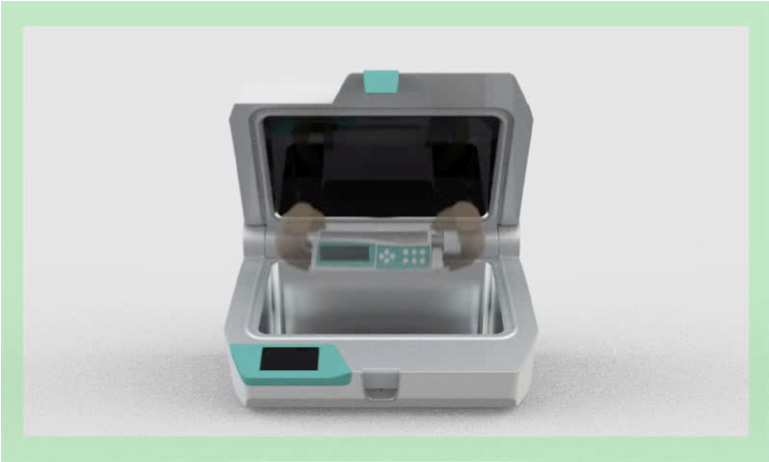

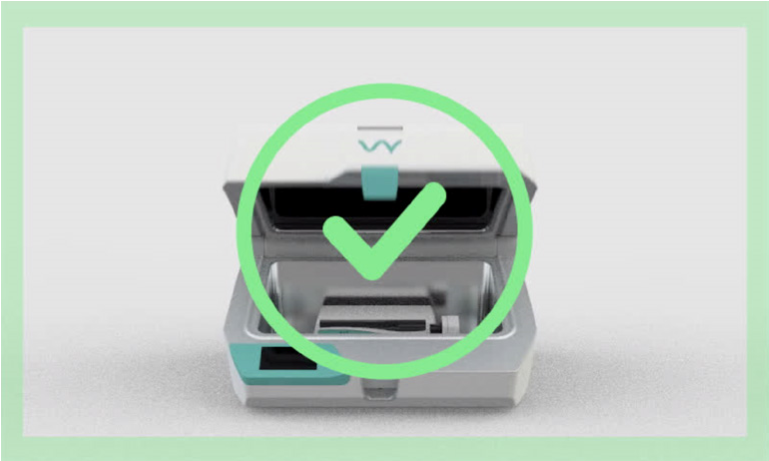


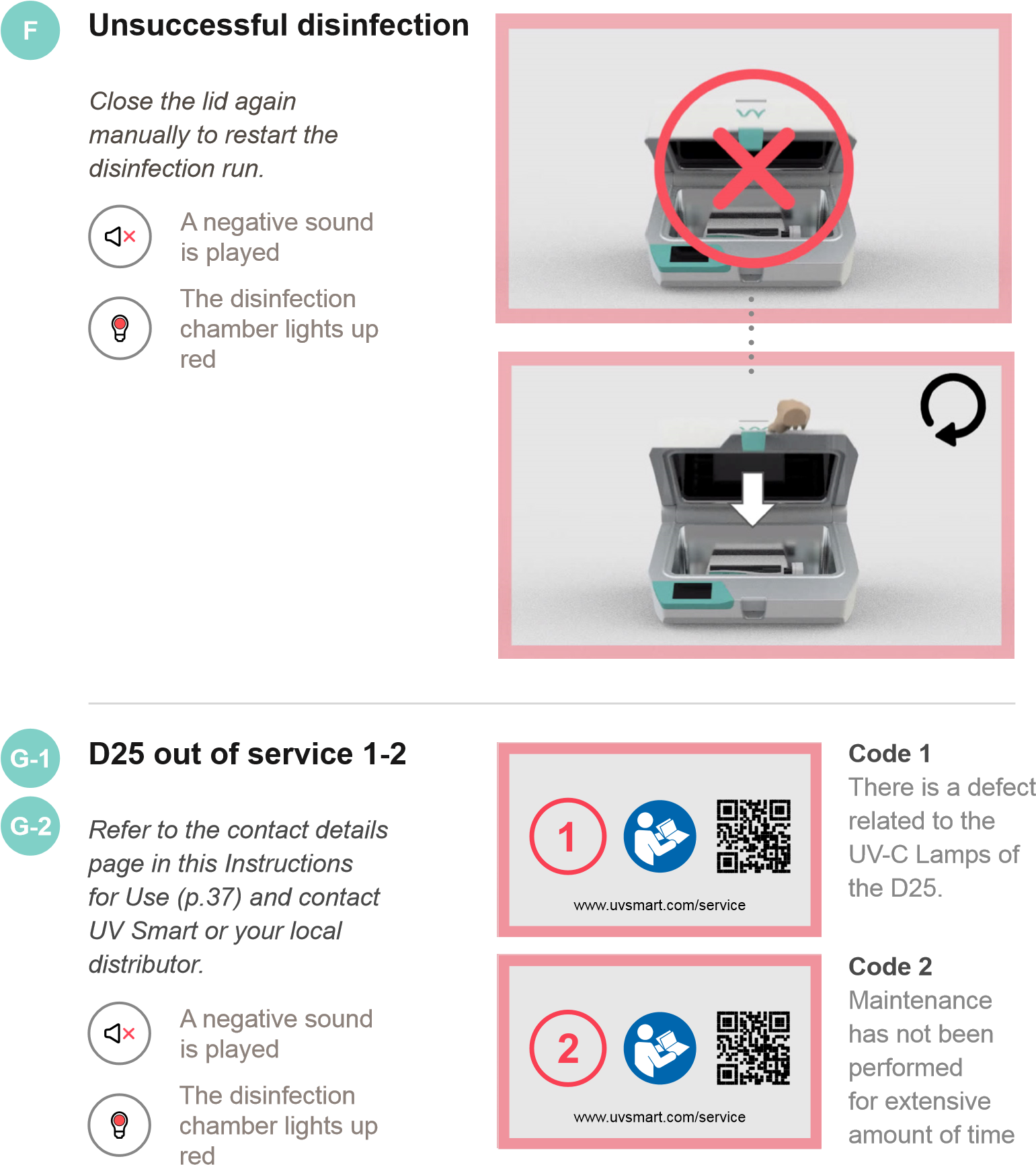


*Use antiseptic wipes to clean*

*the following features:*

•

*Upper and lower*

*disinfection chamber*

*(walls and glass plates)*

•

*Top surface and display*

•

*Handle*

*Close the lid manually after*

*cleaning. Refer to page 28 in*

*this Instructions for Use for*

*more information on cleaning.*

**Manual D25 cleaning**

**H**

An attention sound

is played

An attention sound

is played

The disinfection

chamber blinks

white light

The disinfection

chamber blinks

white light

**!**

**!**


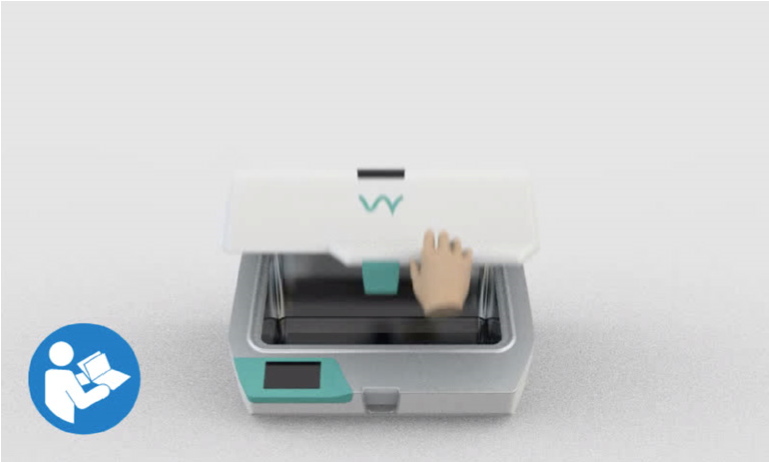

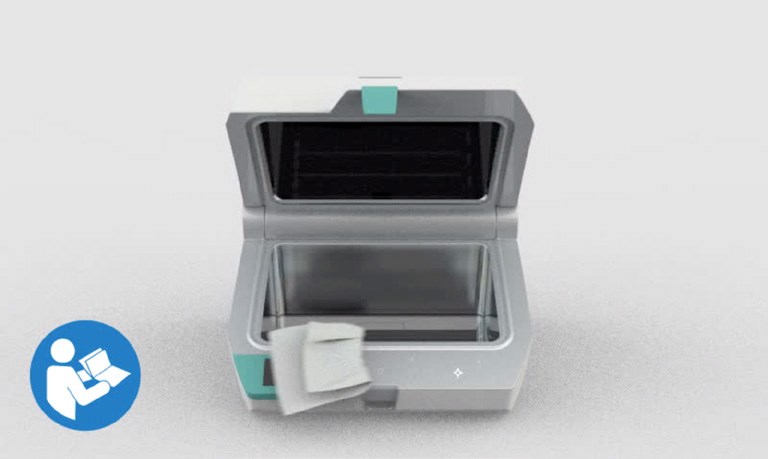

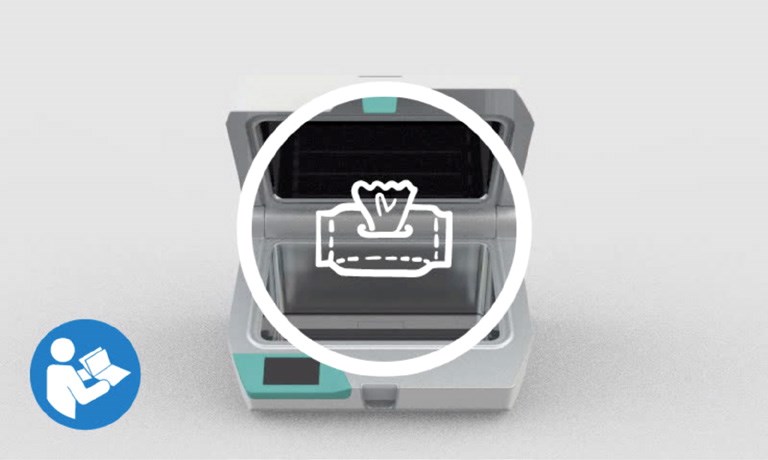

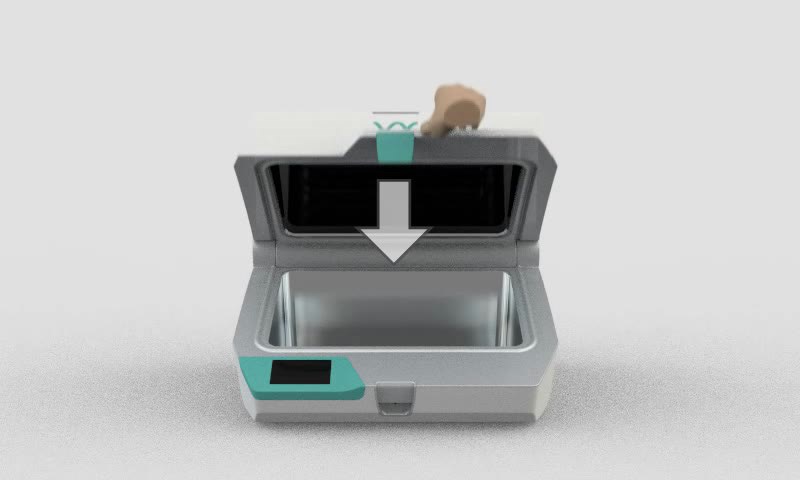


*Open the lid manually by*

*pulling the clip*

**Open the lid manually**

**I**


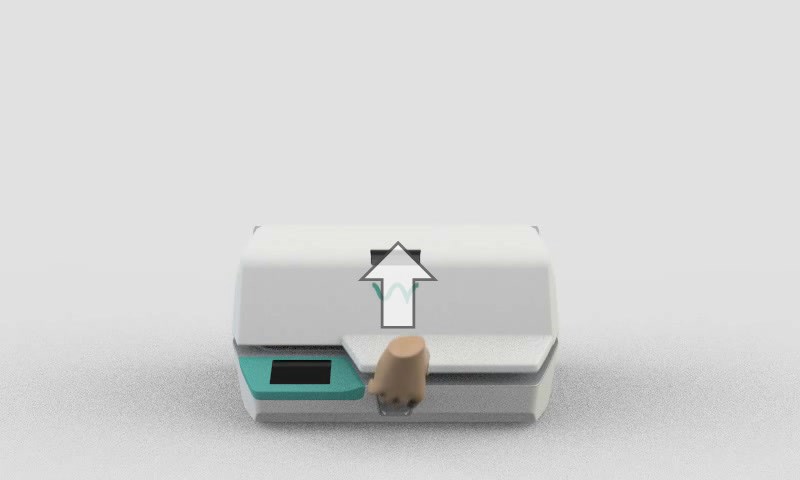


# Maintenance

### Cleaning

The UV Smart D25 is a multiple use device and its surfaces should only be cleaned with antiseptic wipes. After cleaning, allow the D25 to vent for at least 1 minute prior to use. Do not use spray disinfectants into the openings of the D25 or at the mains power connection. Do not use agressive cleaning agents like bleach, paint thinner, acetone or acids.

**1**

*Clean the bottom*

*disinfection chamber*

*(*

*walls and glass plate)*

*with an antiseptic wipe.*

*Clean the top*

*disinfection chamber*

*(*

*walls and glass plate)*

*with an antiseptic wipe.*

**2**


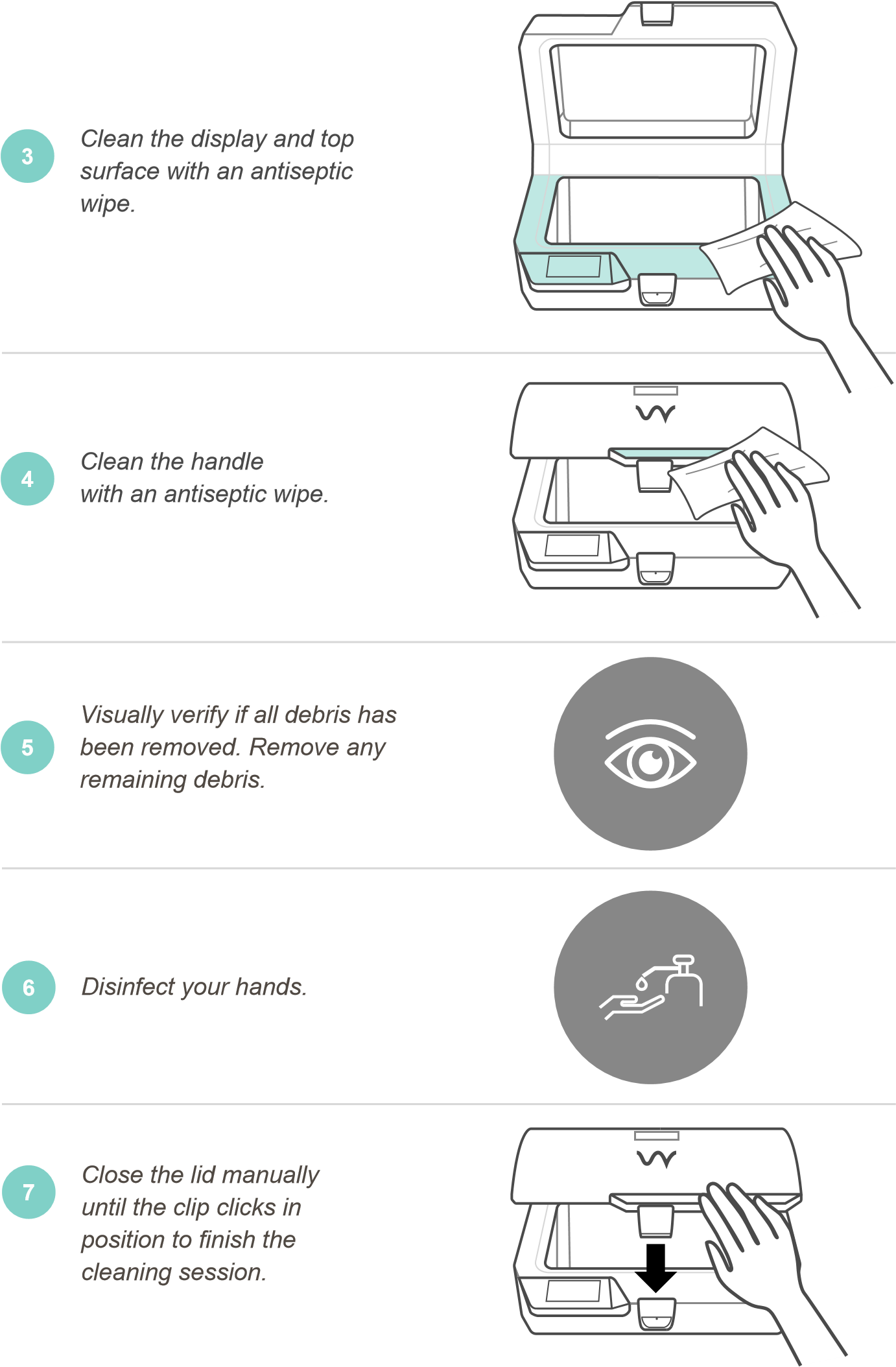


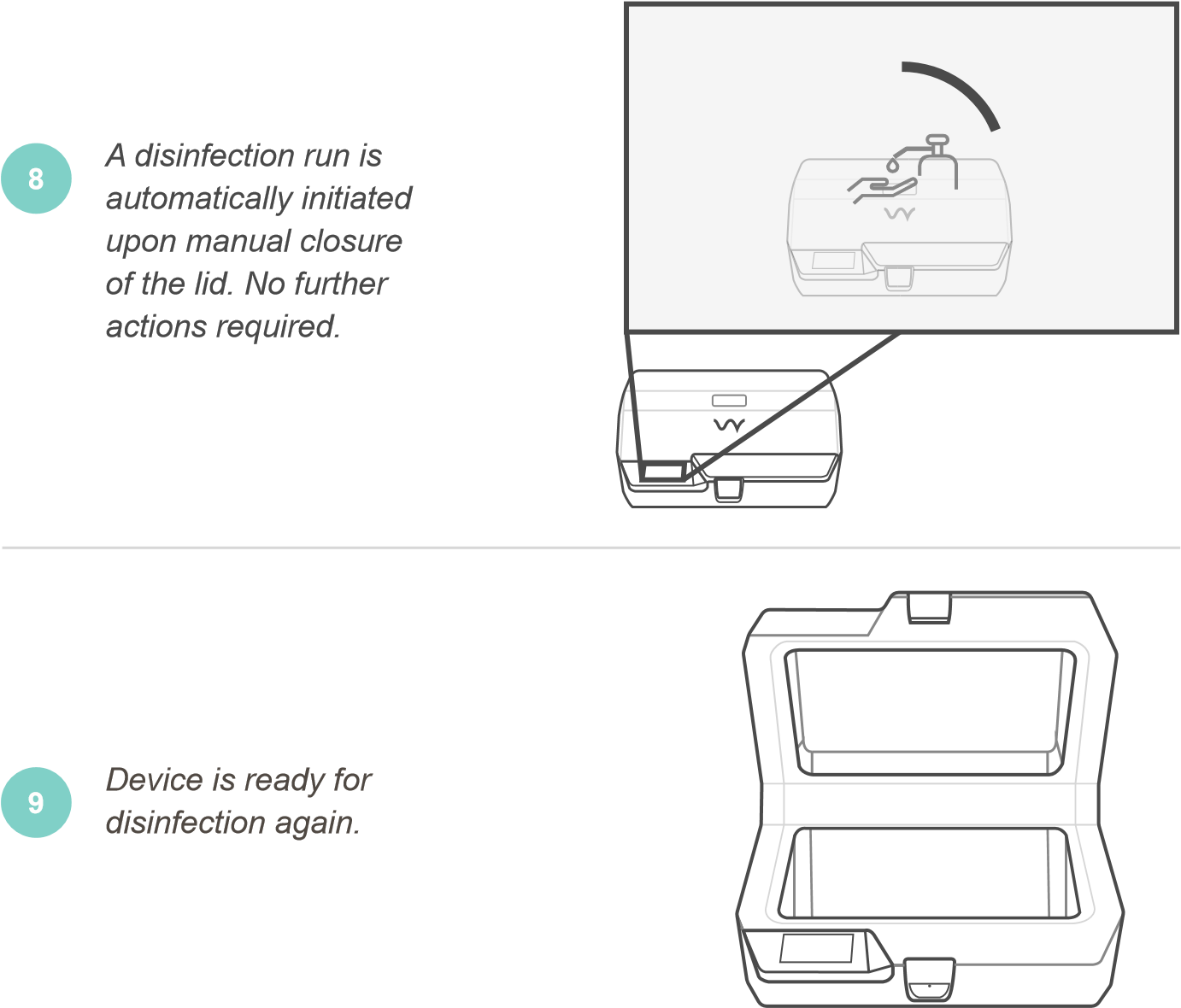


### Transport

**1**

*Switch off the D25.*

**Off**

**On**


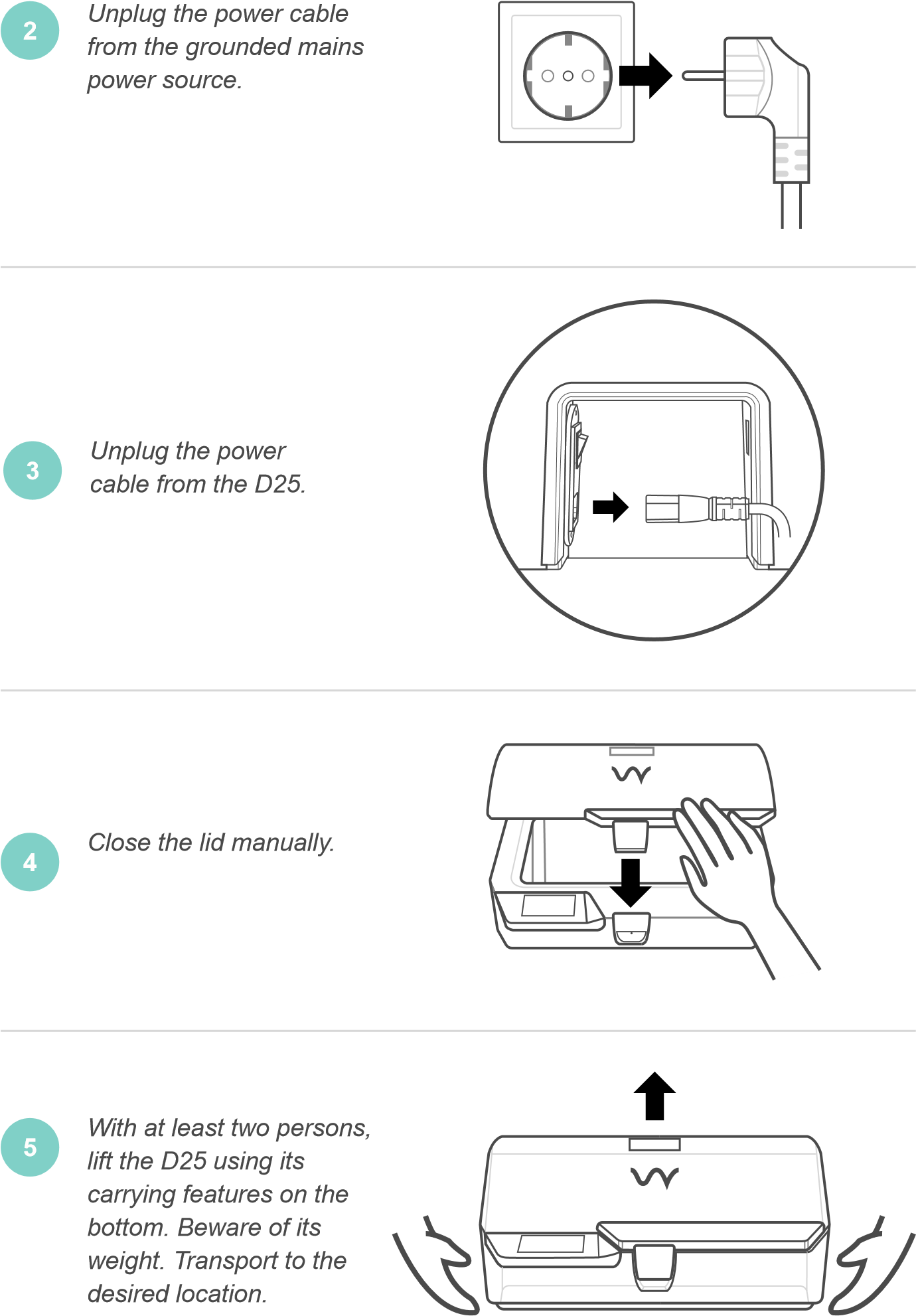


## Technical safety check

Periodic servicing should be performed on the D25 based on the service agreement. The technical safety check is recommended to be carried out every year and should be documented. This check should be carried out exclusively by qualified personnel. Contact UV Smart or your local distributor for further information.

### Disposal

The UV Smart D25 can be returned to UV Smart for further disposal. Please contact UV Smart or your local distributor for further information.

#
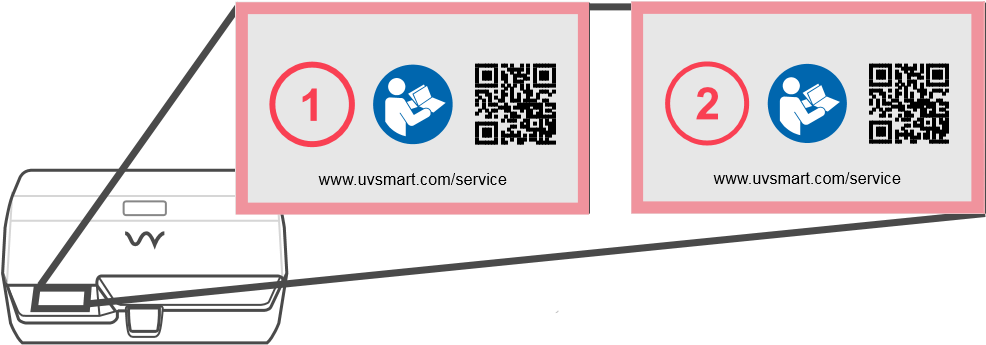
Problem solving

### Problem solving

In case of an occurring problem, please refer to the interface pages (21-27) and identify the meaning behind light, sound & display feedback and take corresponding action. Contact UV Smart or your local distributor in case you were not able to resolve the problem.

### Error codes

The D25 can show two types of error codes, each referring to a specific issue. Contact UV Smart or your local distributor with the corresponding code to resolve

the issue.

#### Code 1 Code 2

There is a defect Maintenance has not

related to the UV-C been performed for

Lamps of the D25. extensive amount of time.

## Broken glass or lamps


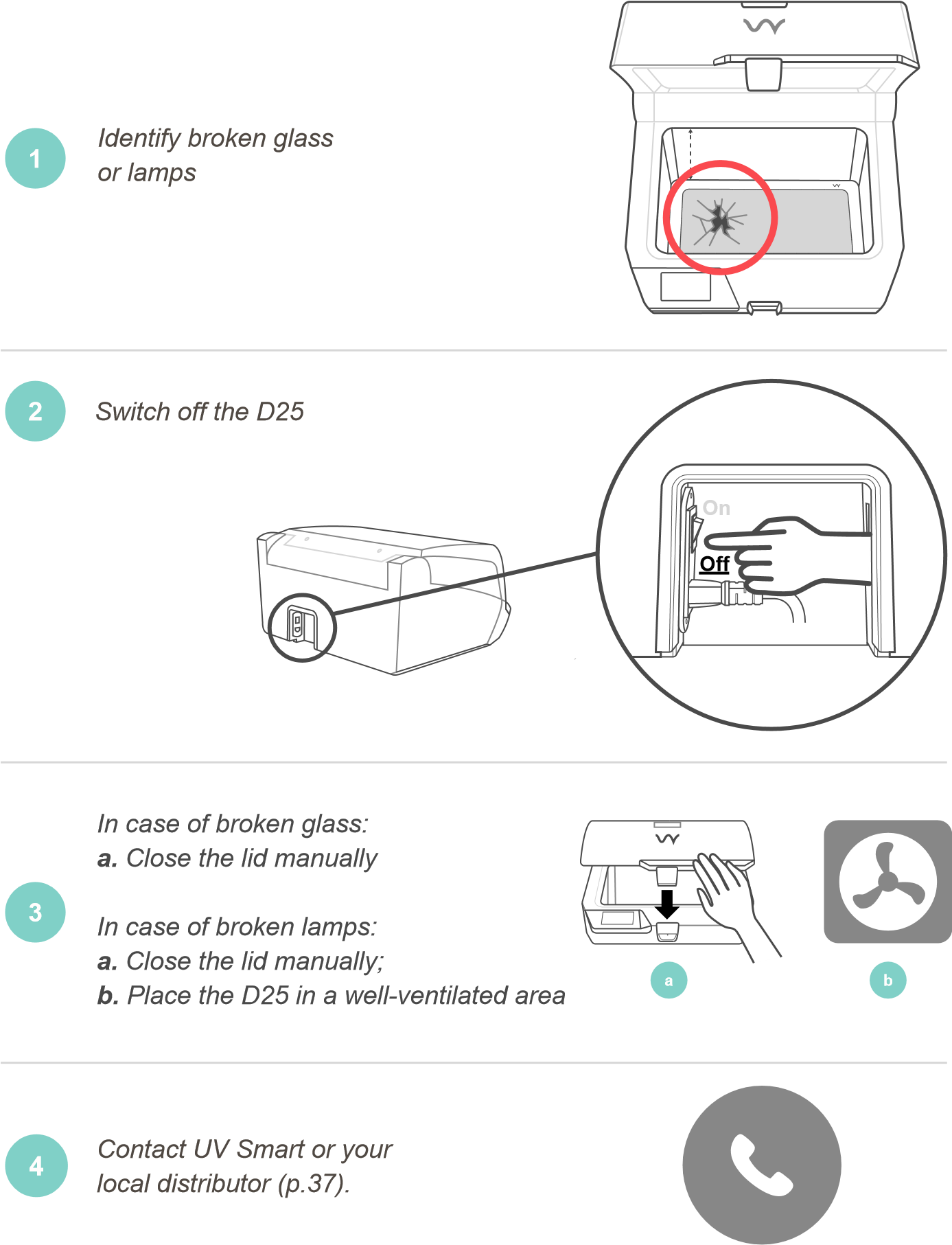


### Overheating

In case of the D25 overheating, unplug the power cable from the mains power. In case of a fire, extinguish it with a fire extinguisher. Call UV Smart or your local distributor to report and resolve any issue.

# Technical specifications

Type of unit D25 UV-C Medical Disinfection Device

| Classification (acc. to IEC/EN 60601-1) | Protective Class I |
| --- | --- |
| Class (acc. to Directive 93/42/EEC) | IIa |
| Ingress Protection | IP 21 |
| Voltage rating | 220-240VAC |
| Frequency | 50/60Hz |
| Amperage rating | 2.5A |
| Max. power rating | 220W |
| UV-C range | 100-280nm (peak at 253.7nm) |
| Accessible fuse value | 2x1A 250V time-lag fuse (ø5x20mm) |
| EMC | IEC/EN 60601-1-2 / 61000-3-2 / 61000-3-3 |
| Product lifetime | 5 years |
| Operating conditions  Relative humidity  Temperature  Atmospheric pressure | 20% - 90% (without condensation)  -20°C - +50°C  700hPa - 1060hPa |
| Transport and storage conditions  Relative humidity  Temperature  Atmospheric pressure | 20% - 90% (without condensation)  -20°C - +50°C  700hPa - 1060hPa |
| Weight | Approx. 25kg |
| Dimensions (W x H x D) | 557mm x 589mm x 489mm (open)  557mm x 297mm x 489mm (closed) |
| Computer connection | USB |
| Historical data | Retrievable via UV Smart - log clearing after maintenance |

Technical inspection (safety check) Every 12-18 months

# Symbols

| **MD** |
| --- |


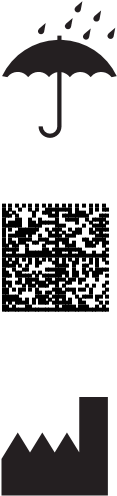
Keep dry


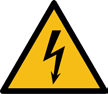

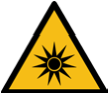


Protect the medical equipment from moisture.

GS1 Data Matrix Data Matrix containing the unique Device Identification.

Legal Manufacturer Entity responsible for design & manufacturing of the product.

IP21*Ingress Protection Classification*

*Degree of protection of electrical*

*appliances against the intrusion of water, solid objects and dust.*

*Temperature limits Indicates the lower and upper temperature limits.*

*CE Marking + Notified Body number*

*The product is CE-certified by*

**2274** *Notified Body 2274.*

*Medical Device*

*The product is a medical device.*

*Alternating current input The equipment is suitable for alternating current only.*

*Accessible fuse value Value of the fuse(s) present in the product.*

*Optical radiation*

*Warning of optical radiation.*

Mandatory to read the Instructions for Use

The Instructions for Use must be read.

Caution

Consult the Instructions for Use for important information.

Date of Manufacture Indicates the date on which the product was manufactured.

*REF-number*

*Catalogue reference number.*

*Atmospheric pressure limits Indicates the lower and upper atmospheric pressure limits.*

*Protective earth (ground) Protection against electrical shock in case of a fault.*

*Humidity limits Indicates the lower and upper humidity limits.*

*SN-number*

*Serial number symbol.*

*Non-ionizing The product emits non-ionizing radiation.*

*WEEE-symbol*

*Waste Electrical and Electronic equipment.*

*Maintenance Maintenance should be performed. Contact UV Smart.*

*Dangerous voltage Indicates hazards arising from dangerous voltages.*

# Warranty

UV Smart provides 24 months warranty as from the date of delivery, for every UV Smart D25. This covers repair or replacement of parts damaged as a result of design/manufacturing errors or material defects. It is not allowed to perform any modifications or repairs per customer’s decision. Modifications or repairs to the unit undertaken by the user/operator or by third parties invalidate the warranty.

The warranty does not cover the following:

▪ Elimination of faults attributable to incorrect/unauthorized handling, or to abnormal wear and tear.

# Contact Details

For any problems or questions please contact UV Smart or your local distributor.

**UV Smart B.V.** +31 850 609 800 info@uvsmart.nl

Olof Palmestraat 16

2616LR Delft

The Netherlands

*This page has been left blank intentionally.*

*This page has been left blank intentionally.*


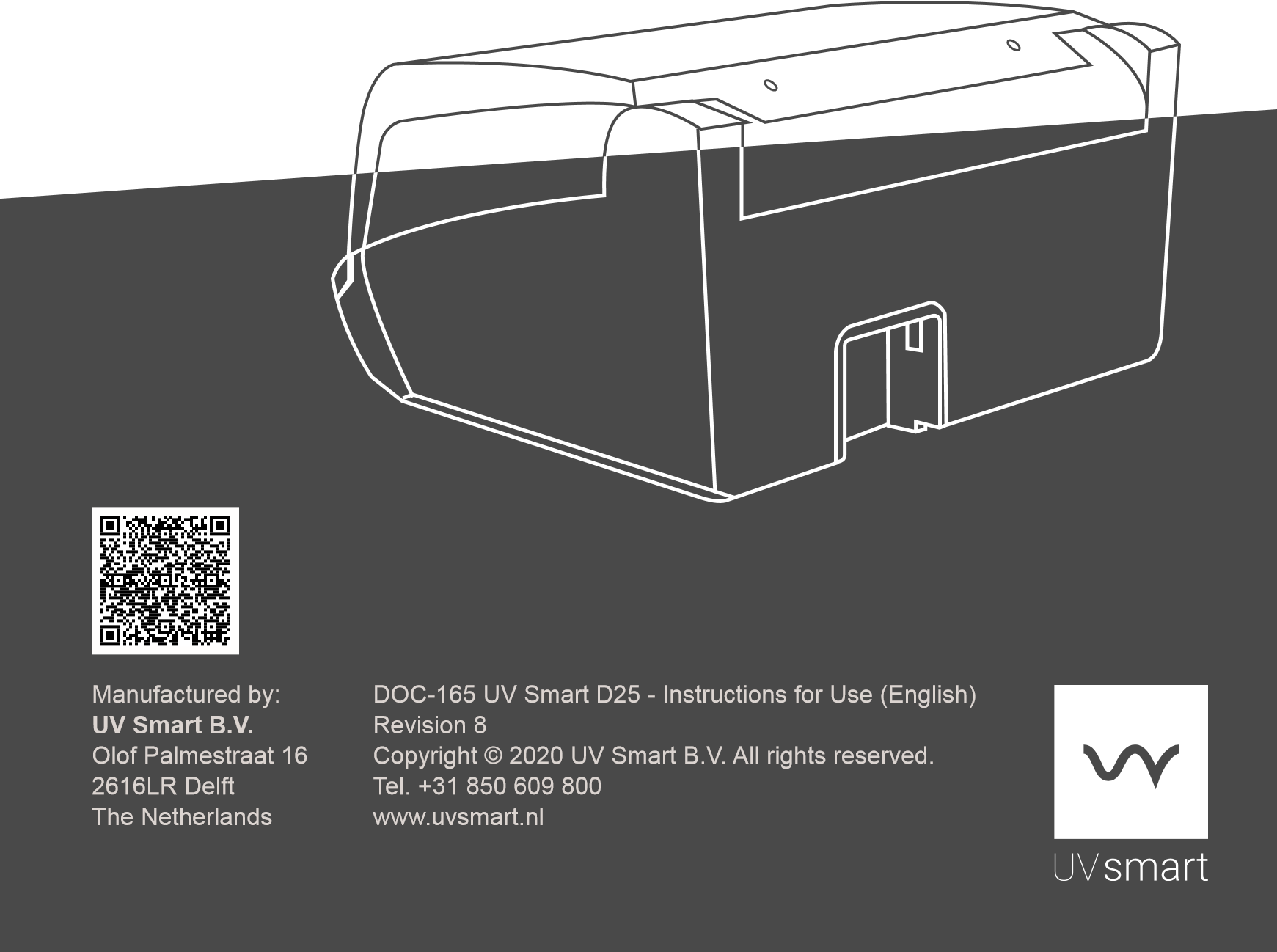

Supplement: Multimedia component 1 [file mmc1.docx]
